# Decomposing mental health inequities: an Oaxaca-Blinder analysis of ethnic and gender disparities in the UK

**DOI:** 10.1186/s12939-026-02980-2

**Published:** 2026-08-17

**Authors:** Facundo Herrera, Giuseppe Migali, Céu Mateus

**Affiliations:** Division of Health Research, Lancaster University, Lancaster, UK

## Abstract

**Background:**

Ethnic minority women in the United Kingdom experience worse mental health than the general population, yet the mechanisms driving this gap remain unclear. This study examines how much of the gap is explained by differences in social determinants and how much reflects differential returns to those determinants across groups.

**Methods:**

We used pooled data from 14 waves of the UK Household Longitudinal Study (2010–2024), comprising 291,711 person-wave observations from 50,336 unique individuals. We compared ethnic minority women with the rest of the UK adult population. Mental health was measured using the SF-12 Mental Component Summary and the GHQ-12 Likert scale. Oaxaca-Blinder decomposition with pooled coefficients separated the observed gap into an explained component, mapping to differential exposure in the Diderichsen framework, and an unexplained component, which contains both differential vulnerability and unmeasured factors. A within-women contrast was estimated as a co-equal main analysis, comparing ethnic minority women with white women only. Sensitivity analyses tested the robustness of results to carry-forward imputation, income specification, the inclusion of limiting illness, and within-person clustering. Secondary analyses examined ethnicity without gender and disaggregated ethnic groups.

**Results:**

Ethnic minority women scored 1.83 points lower on the SF-12 (*p* < 0.001) and 0.81 points higher on the GHQ-12 (*p* < 0.001) than the rest of the population. A substantial share of the SF-12 gap was associated with differences in group characteristics, primarily age composition, home ownership, marital status, and neighbourhood cohesion, although the explained share varied across specifications (38% to 91% across the sensitivity analyses). The overall unexplained component was non-significant but masked substantial opposing effects: education was less protective of functional mental health for ethnic minority women, while home ownership was more protective. These differential vulnerability effects reversed between the SF-12 and the GHQ-12. The within-women contrast yielded a non-significant raw gap (0.60 points, *p* = 0.13) but a similar pattern of compositional disadvantage offset by differential returns. The mental health gap was not visible when ethnicity was examined without gender. Disaggregated analyses revealed distinct patterns: for Asian women, the GHQ gap was driven almost entirely by differential vulnerability, while for Black women, substantial compositional disadvantage was offset by protective differential returns.

**Conclusions:**

The mental health gap between ethnic minority women and the rest of the UK population is substantial, and a large share is associated with differences in observed characteristics, notably age composition, home ownership, marital status, and neighbourhood cohesion. The unexplained component is sensitive to specification and should not be read as a clean estimate of differential vulnerability. Significant differential returns operate in opposing directions beneath the surface. The findings are consistent with two complementary policy directions: redistributing access to the social determinants associated with much of the observed gap, and addressing the structural conditions under which those determinants translate into mental health returns.

**Supplementary Information:**

The online version contains supplementary material available at https://doi.org/10.1186/s12939-026-02980-2.

## Background

Ethnic minority women in the United Kingdom experience worse mental health than the general population. These disparities persist despite decades of policy attention and reflect the compounding effects of racial and gender disadvantage [1, 2]. Yet the mechanisms driving these gaps remain poorly understood. Ethnic minority women may face more adverse social conditions, or the same conditions may affect them differently, or both. Distinguishing between these pathways matters for policy. The first calls for redistributing resources; the second requires tackling structural discrimination. This study uses Oaxaca-Blinder decomposition to disentangle these mechanisms, drawing on 14 waves of the UK Household Longitudinal Study.

Ethnic minorities in the UK have poorer mental health than the white British majority. Researchers have documented these inequalities for over fifty years across prevalence, access, treatment quality, and outcomes [3–5]. Black Caribbean and Black African individuals face elevated risks of psychosis-related diagnoses and higher rates of detention under the Mental Health Act [6]. Ethnic minorities more often enter mental health services through crisis pathways than primary care [7]. The Mental Health Bill introduced in 2024 acknowledges ongoing ethnic disparities in detention rates. Despite growing evidence, research on ethnic health inequalities in the UK remains sparse compared to other dimensions of disadvantage [2]. The COVID-19 pandemic renewed attention to these gaps, as ethnic minorities experienced worse impacts on both physical and mental health [8]. Using UKHLS data, Quintana-Domeque and Proto [9] found that women of all ethnic backgrounds saw greater mental distress during the pandemic than white British men. Ethnic minority men showed a similar pattern.

Discrimination is a central pathway linking ethnic minority status to poor mental health. Prospective UK evidence shows clear associations between racial discrimination and adverse health outcomes [10]. Earlier studies established links between racial harassment and psychological distress [11, 12]. The strength of these links varies across generations [13]. Racism operates not as individual prejudice alone but as a system of institutional disadvantage spanning ethnicity, religion, language, and culture [14]. Mental healthcare reflects this systemic character. A meta-ethnography of 66 UK qualitative studies found that narrow models of care act as major barriers, with ethnic minority service users reporting racist practice and dismissal of their experiences [15]. Cultural barriers, language difficulties, and lack of trust in providers reduce uptake of primary mental health services among specific communities [16, 17].

Understanding these inequities requires attention to how multiple axes of disadvantage interact. Crenshaw introduced intersectionality to capture how different forms of oppression operate simultaneously rather than independently [18]. For ethnic minority women, racial and gender discrimination may compound to create mental health challenges distinct from either axis alone. The 2017 Race Disparity Audit identified Black women as the group most likely to experience common mental disorders [1]. Neither ethnicity nor gender alone explains this finding; it reflects their intersection. Qualitative research confirms that young ethnic minority women face intersecting discrimination across schools, workplaces, and public spaces [19]. This generates cumulative stress and distinct mental health difficulties. Despite growing theoretical interest, few studies apply quantitative methods to intersectional health questions. A systematic review found limited quantitative intersectional work on women’s mental health [20]. Bauer and Scheim [21] proposed analytic frameworks for quantitative intersectionality, but applications using decomposition methods remain rare.

The social determinants of health framework, represented by Dahlgren and Whitehead’s rainbow model, describes the layers of influence on population health [22]. However, Dahlgren and Whitehead [23] acknowledged that their model addresses determinants of health rather than mechanisms generating inequities between groups. Diderichsen et al.‘s [24] model fills this gap. It specifies four pathways through which social position produces health inequities: social stratification, differential exposure, differential vulnerability, and differential consequences. Diderichsen et al. [25] further developed these concepts, linking differential vulnerability to mediation and interaction analysis. This framework maps directly onto Oaxaca-Blinder decomposition. The explained component captures differential exposure: it shows how much of the gap arises because ethnic minority women have different levels of social determinants. The unexplained component captures differential vulnerability: whether the same determinants yield different mental health returns across groups. This mapping drives the present study. It allows us to interpret decomposition results within a framework that distinguishes mechanisms requiring different policy responses.

The Oaxaca and Blinder decomposition was developed to analyse wage gaps [26]. Health researchers have increasingly adopted the method to understand mechanisms underlying disparities. Brydsten et al. [27] examined mental health inequalities between native-born and foreign-born individuals in Sweden, finding that social integration factors explained large proportions of observed differences. Allen et al. [28] applied the method to health inequalities across Welsh local authority areas. Meulman et al. [29] extended the approach to socioeconomic differences in self-rated health and healthcare expenditure in the Netherlands, incorporating social determinants from the WHO framework. Jester et al. [30] showed that social determinants underlie racial and ethnic disparities in psychological wellbeing among older adults in the United States. Zheng et al. [31] applied the method to mental health inequalities between migrants and non-migrants in Shanghai. Despite this growing body of work, no study has applied Oaxaca-Blinder decomposition to ethnic and gender mental health inequities using an intersectional grouping, either in the UK or internationally.

This gap matters for three reasons. First, the UK’s ethnic diversity and well-documented mental health disparities make it a critical case for decomposition analysis. Second, existing research has examined ethnicity or gender separately, missing gaps that emerge only at their intersection. Third, 14 waves of UKHLS data, including an ethnic minority boost sample, provide an ideal resource. This study uses data from UKHLS waves 2–15 (2010–2024). We apply Oaxaca-Blinder decomposition to mental health measured by both the SF-12 Mental Component Summary and the GHQ-12. We frame results within Diderichsen et al.‘s model of health inequity mechanisms. Two questions guide the analysis. First, given the gap in mental health between ethnic minority women and the rest of the population, how much of this gap do differences in social determinants explain? Second, how much of the gap reflects differential returns to similar social determinants across groups?

## Methods

### Data and sample

This study drew on data from the UK Household Longitudinal Study (UKHLS), also known as Understanding Society. The UKHLS is a nationally representative panel survey of approximately 40,000 UK households, conducted annually since 2009 [32]. We used waves 2 to 15, covering the period 2010–2024 [33]. Wave 1 was excluded because the required survey weights were unavailable for that wave.

The UKHLS includes an Ethnic Minority Boost (EMB) sample alongside the General Population Sample (GPS). This oversampling of ethnic minority households provides sufficient statistical power to examine health disparities across ethnic groups. Our analytical sample comprised adults aged 16 and over with valid responses on mental health measures and key covariates.

We treated the data as pooled cross-sections rather than a balanced panel. Individuals could therefore contribute observations across multiple waves, maximising sample size. Sample sizes varied by model specification. For the main intersectional model (M1), the SF-12 analytical sample included 291,711 person-wave observations from 50,336 unique individuals, and the GHQ-12 sample included 292,586 observations from 50,417 individuals (see Table 1). Time-varying covariates take their wave-specific value, so a respondent observed in multiple waves contributes one observation per wave, with age, employment, housing tenure, marital status, neighbourhood, and income measured at each wave. This structure is not equivalent to a single cross-sectional dataset, and we address its implications for inference in the Statistical analysis subsection and in the Limitations.

### Outcome measures

We used two validated and complementary measures of mental health available in the UKHLS self-completion questionnaire. The SF-12 Mental Component Summary (MCS) is a continuous measure ranging from 0 to 100, where higher scores indicate better mental health. It is derived from the 12-item Short Form Health Survey and captures general psychological wellbeing, including vitality, social functioning, and emotional role limitations [34]. The 12-item General Health Questionnaire (GHQ-12) was scored using the Likert method (range 0–36), where higher scores indicate greater psychological distress. The GHQ-12 is designed to detect common mental disorders such as anxiety and depression [35].

Using both measures allowed us to capture different dimensions of mental health. The SF-12 MCS reflects broader wellbeing, whilst the GHQ-12 is more sensitive to recent psychological distress.

### Grouping variables

For the primary intersectional analysis, we constructed a binary indicator identifying ethnic minority women (coded 1) versus the rest of the population (coded 0). Ethnicity was classified into five groups based on the UKHLS harmonised variable: White (British, Irish, other White), Mixed, Asian, Black, and Other. These were then collapsed into a binary White/non-White indicator. Sex was coded as female or male. The intersectional grouping variable was defined as the interaction of non-White ethnicity and female sex.

Secondary analyses used alternative grouping variables. Model M4 compared all ethnic minority respondents against White respondents, irrespective of sex. Model M5 disaggregated the intersectional group, comparing Asian women and Black women separately against the rest of the population.

### Explanatory variables

Explanatory variables were selected to align with the Diderichsen et al.’s framework for understanding mechanisms of health inequities. This framework distinguishes between social stratification, differential exposure, and differential vulnerability as pathways through which social position affects health. Variables were operationalised as binary indicators where possible, as this aids interpretation in the Oaxaca–Blinder decomposition. The explanatory variables fall into five domains, described below.

#### Social stratification

Three indicators captured socioeconomic position. Higher education was coded as holding a degree or other higher education qualification. Household income was measured as equivalised net household income, divided by the modified OECD equivalence scale and deflated to constant prices using the Consumer Price Index. For the main models we dichotomised at the survey-weighted median, yielding an indicator for above-median income. For sensitivity model M7 we used the natural logarithm of equivalised income. We discuss the income contribution and the sensitivity comparison in the Sensitivity analyses subsection. Employment status indicated whether the respondent was employed (including self-employment) or in education or training.

#### Differential exposure — material conditions

Home ownership distinguished owner-occupiers from all other tenure types. Rural residence was coded from the UKHLS urban/rural indicator.

#### Differential exposure — psychosocial factors

Marital status indicated whether the respondent was married, in a civil partnership, or cohabiting. Presence of children captured whether any children were living in the household. Neighbourhood cohesion was measured using the Buckner Neighbourhood Cohesion Index, a continuous scale ranging from 1 to 5, with higher scores indicating greater perceived cohesion [36]. Experience of discrimination was a binary indicator coded as 1 if the respondent reported being physically attacked or avoiding places due to fear of attack motivated by race, religion, or gender, in the preceding 12 months. The construct is heterogeneous across groups: for ethnic minority respondents the underlying motivation is more often racial or religious, while for the reference group it is more often gender-related. We address the implications in the Discussion.

#### Health status

Limiting long-standing illness was coded as a binary indicator. Respondents who reported a long-standing health condition and identified at least one functional limitation were coded as 1; all others were coded as 0. We include limiting illness in M1 because it is a strong correlate of mental health and its omission would inflate the unexplained component through omitted-variable bias. We test the over-controlling concern in M8 (see Sensitivity analyses), which drops the variable.

#### Demographics

Age was entered in years alongside a quadratic term (age squared) to capture non-linear associations with mental health.

Sex was included as a covariate in the ethnicity-only model (M4) but was excluded from the intersectional models (M1–M3, M5) to avoid collinearity with the grouping variable.

#### Rotating module variables and carry-forward imputation

Two key variables — neighbourhood cohesion and discrimination — are collected only in selected UKHLS waves as part of rotating modules. Neighbourhood cohesion was measured in waves 1, 3, 6, 9, and 12. Discrimination was measured in waves 1, 3, 5, 7, 9, and 11. To maximise sample coverage, we applied within-person carry-forward imputation. For each individual, missing values were filled first from the most recent prior observation and then, if still missing, from the nearest subsequent observation. This assumes that these characteristics are relatively stable over short intervals between measurement occasions. Within waves 2 to 15, both rotating modules are simultaneously observed only in waves 3 and 9. The observed-only model (M3) therefore draws predominantly on these two waves, which contributes to its smaller and selectively-waved Group 2 sample.

To assess the sensitivity of results to this imputation strategy, we estimated two additional specifications. Model M2 excluded both variables entirely, providing a larger analytical sample (~ 383,000 observations) free of any imputation assumptions. Model M3 retained both variables but used only directly observed values, with no carry-forward imputation (~ 176,000 observations). Comparing results across M1, M2, and M3 allows readers to judge the robustness of findings to these data handling decisions.

For the main analytical sample, 27.6% of discrimination values and 26.5% of neighbourhood cohesion values are directly observed in the wave to which they refer. The remainder are filled by carry-forward (mean wave-distance 2.2 and 1.9 years respectively) or carry-back (1.2 and 1.6 years). Table A19 reports the full breakdown.

### Statistical analysis

#### Oaxaca–Blinder decomposition

Kitagawa showed how a difference in means between two groups can be partitioned into a component attributable to differences in composition and a component attributable to differences in rates [37]. Oaxaca and Blinder extended this to the regression context [38, 39]. We used the Oaxaca–Blinder decomposition to partition mean differences in mental health between groups into two components.

The explained component captures the share of the gap attributable to differences in the distribution of observed characteristics between groups. In the Diderichsen framework, this corresponds to differential exposure: the extent to which groups differ in their levels of social determinants. The unexplained component captures the share attributable to differences in the association between those characteristics and mental health, plus the contribution of unobserved factors. This maps to differential vulnerability: the same characteristic may have a different effect on mental health depending on social position.

The decomposition differs from the mediation approaches more familiar in epidemiology. In a mediation analysis, covariates are added sequentially and attenuation of the association with social position is read as an indirect effect. That approach assumes a causal ordering, in which the mediator lies on the pathway between exposure and outcome. The Oaxaca-Blinder decomposition makes no such assumption. Covariates enter as a single vector with no ordering among them, and the estimates do not change if they are reordered. Each contribution to the explained component shows how much of the gap is associated with a group difference in that characteristic, holding the pooled coefficients fixed. A contribution may therefore be positive or negative. Negative contributions identify characteristics on which the disadvantaged group compares favourably, and which consequently narrow rather than widen the observed gap. The decomposition is an accounting partition of a mean difference, not a causal path model, and we interpret it accordingly. The one place where causal ordering is at issue is limiting long-standing illness, which may lie between social position and mental health. Model M8 addresses this by removing the variable.

We used pooled (Neumark) coefficients as the reference structure for the decomposition [40]. This approach estimates a single set of coefficients from a model combining both groups and uses these as the counterfactual, rather than treating either group’s coefficients as the norm. The pooled specification is recommended when there is no clear basis for choosing one group as the reference, and it provides estimates that are more directly relevant to policy because they reflect the population-average association [41].

We note that individual contributions to the unexplained component are sensitive to the choice of reference category for categorical variables, a well-known issue termed the “transformation problem” [42]. Since all explanatory variables in our specification are binary (with the exception of age, age squared, and neighbourhood cohesion), this concern is substantially reduced but not eliminated. We therefore interpret the detailed unexplained results as indicative of patterns rather than precise point estimates. For binary covariates with symmetric recoding (for example, owns versus rents, or degree versus no degree), the sign of the coefficient and the mean difference both reverse, leaving the contribution to the decomposition unchanged [43]. The remaining sensitivity is therefore confined to the constant and to truly multi-category variables, and is small in our specification.

#### Survey design and estimation

All analyses incorporated the UKHLS complex survey design. We specified primary sampling units, stratification variables, and probability weights using Stata’s survey estimation framework. We used the individual-level longitudinal self-completion weight (indscus_lw) for both the GPS and EMB samples. This weight calibrates to the longitudinal respondent population at each wave, conditional on prior-wave participation, and adjusts for unequal selection probabilities, differential non-response, and known population totals. Cross-sectional weights are not available in the protected data file, so no single weight is exactly aligned with a pooled cross-sectional design that combines waves 2 to 15.

This weight adjusts for unequal selection probabilities, differential non-response, and calibrates to known population totals [44].

Decompositions were estimated using the user-written **oaxaca** command in Stata with the svy and pooled options [41]. Standard errors account for stratification and clustering. UKHLS samples individuals geographically, so respondents who contribute multiple waves remain in the same PSU, and PSU clustering therefore absorbs most within-person dependence as well as spatial selection. We confirm this empirically in M9, which re-estimates M1 with clustering at the individual level and produces essentially identical standard errors. Intra-class correlations from random-intercept models are substantial for slowly-changing covariates and for the outcomes (SF-12 0.52, GHQ-12 0.48, education 0.93, home ownership 0.87; full set in Table A18).

#### Model hierarchy

We estimated nine models to examine the mental health gap from different perspectives. Each model was run separately for the SF-12 MCS and GHQ-12 outcomes.

Model M1 (main intersectional) compared ethnic minority women against the rest of the population, including all explanatory variables with carry-forward imputed values for neighbourhood cohesion and discrimination. Model M2 (sensitivity — reduced) used the same grouping but excluded neighbourhood cohesion and discrimination, providing a larger sample without imputation assumptions. Model M3 (sensitivity — observed only) retained both variables but restricted to directly observed values without carry-forward imputation.

Model M4 (secondary — ethnicity only) compared all ethnic minority respondents against White respondents, irrespective of sex, and included sex as an additional covariate. Model M5 (secondary — disaggregated) repeated the intersectional comparison separately for Asian women and Black women against the rest of the population, testing for heterogeneity within the aggregate ethnic minority group.

Model M6 (within-women contrast) compared ethnic minority women against white women only, holding gender constant. We treat M1 and M6 as co-equal main analyses that answer different intersectional questions, drawing on McCall’s distinction between intercategorical and intracategorical analysis and on Bauer and Scheim’s framework for quantitative intersectionality [21, 45, 46]. Three further sensitivity analyses test specific concerns. Model M7 replaces the binary above-median income indicator with the natural logarithm of equivalised household income, addressing the reviewer concern that median-splitting may obscure the income gradient. Model M8 drops limiting long-standing illness from the right-hand side, addressing the over-controlling concern that physical health may lie on the causal pathway between social position and mental health. Model M9 re-estimates M1 with standard errors clustered at the individual level rather than the primary sampling unit, providing an empirical check on within-person dependence. M6 is reported in Decomposition of the within-women contrast; M7, M8, and M9 are reported in Sensitivity analyses.

#### Software

All analyses were conducted using Stata version 18.5. Statistical significance was assessed at the 5% level. Given the large sample sizes, we emphasised effect sizes and practical significance alongside statistical tests when interpreting results.

## Results

### Sample characteristics

The analysis sample comprised 291,711 person-wave observations from 50,336 unique individuals across waves 2 to 15 (mean 5.8 waves per person, range 1 to 14), of which 36,839 person-waves were from ethnic minority women and 253,625 from the rest of the population. Table A20 reports the wave-contribution distribution. Table 1 presents the survey-weighted characteristics of both groups. After excluding wave 1 records (50,994; longitudinal weight unavailable), records with missing outcome (68,683 for SF-12, 60,258 for GHQ), records with missing grouping variable (418 and 422 respectively), and final listwise drops on the M1 covariate vector, the analytical samples comprised 291,711 person-wave observations for SF-12 and 292,586 for GHQ. Table A15 sets out the full attrition flow for both outcomes.

Sample sizes for other models varied according to the variables included (see Methods, Statistical analysis subsection). Table A1 in the Additional file 1 presents descriptive statistics disaggregated by ethnic group.

**Table 1:** Descriptive statistics

*N*	Total sample	Rest of population	Ethnic minority women
	291,711	253,625	36,839
	*Mean (SD) or %*	*Mean (SD) or %*	*Mean (SD) or %*
**Demographics**			
Age (years)	47.7 (18.7)	48.6 (16.6)	39.9 (10.3)
**Social and economic characteristics**			
Married or cohabiting	58.8	59.9	49.3
Children in household	32.1	30.0	50.5
Above median household income	62.1	63.4	49.7
Employed or student	69.5	68.8	74.9
Higher education	41.2	40.5	47.2
Home owner	69.2	70.5	57.4
Rural residence	20.9	22.7	4.8
**Psychosocial factors**			
Experienced discrimination	21.6	21.2	24.4
Neighbourhood cohesion (1–5)	3.5 (0.7)	3.5 (0.7)	3.4 (0.5)
**Health status**			
Limiting long-standing illness	21.8	22.3	17.4
**Outcome measures**			
SF-12 Mental Health (0–100)	48.2 (10.6)	48.4 (9.2)	46.1 (7.6)
GHQ-12 Likert (0–36)	11.4 (5.7)	11.3 (4.9)	12.3 (4.4)

Note: Complete case analysis was used. Missingness was concentrated in the rotating module variables (neighbourhood cohesion and discrimination), which are available in selected waves only. Sensitivity models M2 and M3 address this (see Sensitivity analyses and Methods)

Ethnic minority women were substantially younger than the rest of the population (mean age 39.9 versus 48.6 years). They were less likely to own their home (57.4% versus 70.5%) and far less likely to live in rural areas (4.8% versus 22.7%). Income disadvantage was also evident, with 49.7% of ethnic minority women living in households above the weighted median income, compared with 63.4% of the rest of the population. Rates of limiting long-standing illness were lower among ethnic minority women (17.4% versus 22.3%), reflecting their younger age profile. Discrimination exposure was slightly higher (24.4% versus 21.2%), and neighbourhood cohesion marginally lower (3.4 versus 3.5 on a 1–5 scale).

Educational attainment and employment rates were broadly similar between groups. Ethnic minority women were somewhat more likely to hold higher education qualifications (47.2% versus 40.5%) and to be employed or in education (74.9% versus 68.8%). Family structure differed notably: ethnic minority women were less likely to be married or cohabiting (49.3% versus 59.9%) but more likely to have children in the household (50.5% versus 30.0%).

### Mental health gaps across comparison groups

As set out in Section “Statistical analysis”, we estimated nine models to examine the mental health gap from different angles:

- Main intersectional model (M1): ethnic minority women versus the rest of the population, including discrimination and neighbourhood cohesion (*N* = 291,711).
- Reduced model (M2): as above, but excluding discrimination and neighbourhood cohesion to test sensitivity to carry-forward imputation (*N* = 383,362).
- Observed-only model (M3): as the main model, but restricted to waves in which discrimination and neighbourhood cohesion were directly measured (*N* = 175,779).
- Ethnicity-only model (M4): all non-white versus white respondents, to test whether the gap exists along ethnicity alone (*N* = 300,321).
- Disaggregated model (M5): Asian women and Black women examined separately, to address within-group heterogeneity.
- Within-women contrast (M6): ethnic minority women versus white women only (*N* = 151,770), reported as a co-equal main analysis.
- Continuous log income (M7): as M1 but with log equivalised household income in place of the binary indicator (*N* = 277,018).
- No limiting long-standing illness (M8): as M1 but with limiting illness dropped from the right-hand side (*N* = 291,711).
- Within-person clustering (M9): as M1 but with standard errors clustered at the individual level (*N* = 291,711).

On the SF-12 mental component, ethnic minority women scored 1.83 points lower than the rest of the population (46.02 versus 47.85, *p* < 0.001). The corresponding GHQ-12 gap was 0.81 points (12.35 versus 11.54, *p* < 0.001), with higher scores indicating greater psychological distress.

These gaps were not visible when ethnicity was examined without reference to gender. The raw SF-12 gap between all non-white and white respondents was just 0.19 points and non-significant (*p* = 0.531). The GHQ gap was similarly negligible (0.03 points, *p* = 0.857). The mental health inequity emerged only at the intersection of ethnicity and gender.

Disaggregating the ethnic minority women category revealed further heterogeneity. Asian women showed a significant SF-12 gap of 1.45 points (*p* = 0.003) and a GHQ gap of 0.71 points (*p* = 0.007). Black women showed smaller, non-significant gaps on both measures (SF-12: 0.56, *p* = 0.423; GHQ: −0.04, *p* = 0.929).

### Decomposition of the intersectional gap

The Oaxaca-Blinder decomposition separates the observed gap into two parts. The explained component captures differences in the characteristics of each group — for example, differences in home ownership rates. The unexplained component captures differences in the returns to those characteristics — for example, whether home ownership confers the same mental health benefit for both groups. In the Diderichsen framework, these correspond to differential exposure and differential vulnerability, respectively. Full decomposition results for all models are provided in Additional file 1.

#### SF-12 mental health

**Fig. 1:**
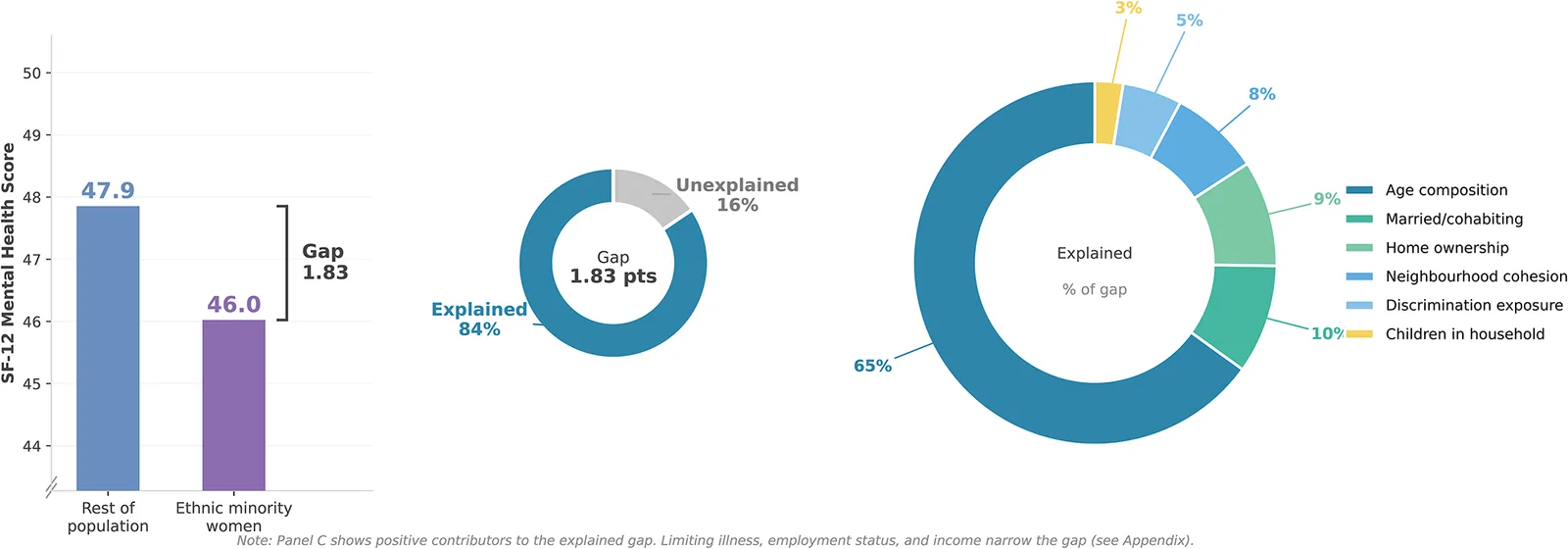
Decomposition of SF-12 mental health gap: ethnic minority women vs. rest of the UK population. Source: UKHLS Waves 2–15 (2010–2024) [33]

The total SF-12 gap between ethnic minority women and the rest of the population was 1.83 points (*p* < 0.001). Of the 1.83 points, 1.55 (85% in M1) were attributable to differences in group characteristics, the explained component (*p* < 0.001). This share is sensitive to specification: it falls to 38% in the observed-only model (M3) and rises to 86% with continuous log income (M7). We discuss the sensitivity in the Sensitivity analyses section. The remaining 0.28 points (15%) were unexplained (*p* = 0.417), meaning they could not be accounted for by compositional differences alone. Figure 1 summarises the overall decomposition and Table A2 in the supplementary material presents the full results.

#### Explained component (differential exposure)

The 1.55 explained points were distributed across the covariates as follows. Age composition was the largest single contributor. The net effect of age and age-squared was + 1.33 points, reflecting the younger age profile of ethnic minority women and the established U-shaped relationship between age and mental health. Lower rates of home ownership contributed + 0.19 points (*p* < 0.001), lower neighbourhood cohesion + 0.16 points (*p* = 0.011), and higher discrimination exposure + 0.11 points (*p* = 0.020). Lower rates of marriage or cohabitation contributed + 0.20 points (*p* < 0.001), comparable to the home ownership contribution and larger than the contribution of discrimination. Two factors worked in the opposite direction, narrowing the gap: lower rates of limiting long-standing illness (− 0.25 points, *p* < 0.001) and higher employment participation (− 0.10 points, *p* = 0.001). These negative values indicate characteristics that, if anything, should give ethnic minority women better mental health than the rest of the population.

#### Unexplained component (differential vulnerability)

**Fig. 2:**
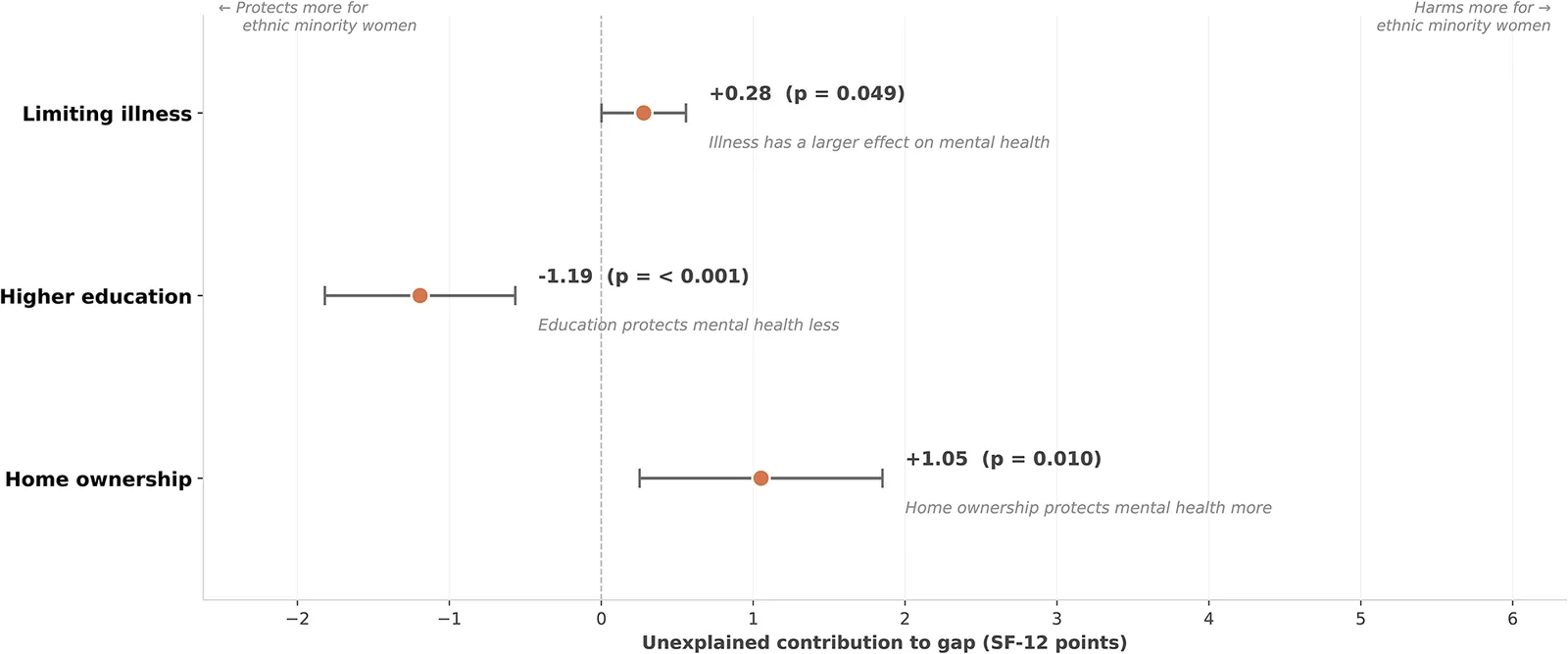
Significant differential vulnerability effects (SF-12). Source: UKHLS Waves 2–15 (2010–2024) [33]

The 0.28 unexplained points were not statistically significant as a whole, but this masked substantial offsetting effects among individual variables (Fig. 2). Each variable’s unexplained contribution captures whether the mental health return to that characteristic differs between groups.

Education showed the largest differential effect. Holding a higher education qualification was associated with a smaller improvement in SF-12 scores for ethnic minority women than for the rest of the population (− 1.19 points, *p* < 0.001). In other words, education was less protective of functional mental health for this group.

Home ownership showed the opposite pattern. Owning one’s home was associated with a greater mental health benefit for ethnic minority women (+ 1.05 points, *p* = 0.010). Limiting long-standing illness also showed a differential effect: illness was associated with a somewhat larger reduction in SF-12 scores for ethnic minority women (+ 0.28 points, *p* = 0.049).

These opposing effects — education protecting less (− 1.19) but ownership protecting more (+ 1.05) — largely cancelled each other out. This is why the overall unexplained component summed to just 0.28 points despite meaningful differential vulnerabilities operating beneath the surface.

#### GHQ-12 psychological distress

**Fig. 3:**
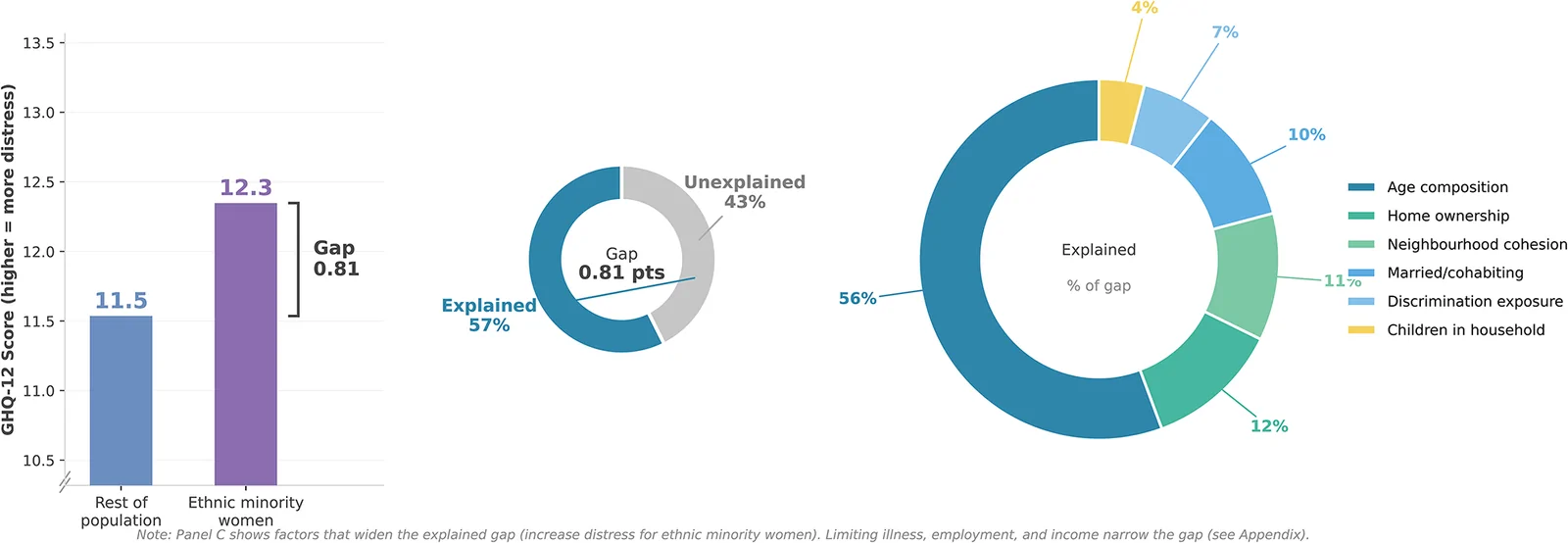
Decomposition of GHQ-12 psychological distress gap: ethnic minority women versus rest of the UK population. Source: UKHLS Waves 2–15 (2010–2024) [33]

The total GHQ-12 gap was − 0.81 points (*p* < 0.001), with ethnic minority women scoring higher (indicating more distress). Of this gap, − 0.47 points (57%) were explained by differences in group characteristics (*p* < 0.001). The remaining − 0.35 points (43%) were unexplained, falling short of conventional significance (*p* = 0.085). Figure 3 summarises the overall decomposition. Full results are presented in Table A3.

#### Explained component

The composition of this component was similar to the SF-12. Age was the dominant factor (net − 0.42 points), followed by home ownership (− 0.09 points, *p* < 0.001) and neighbourhood cohesion (− 0.09 points, *p* = 0.011). As with the SF-12, lower rates of illness (+ 0.16 points, *p* < 0.001) and higher employment (+ 0.06 points, *p* = 0.001) narrowed the gap.

#### Unexplained component

**Fig. 4:**
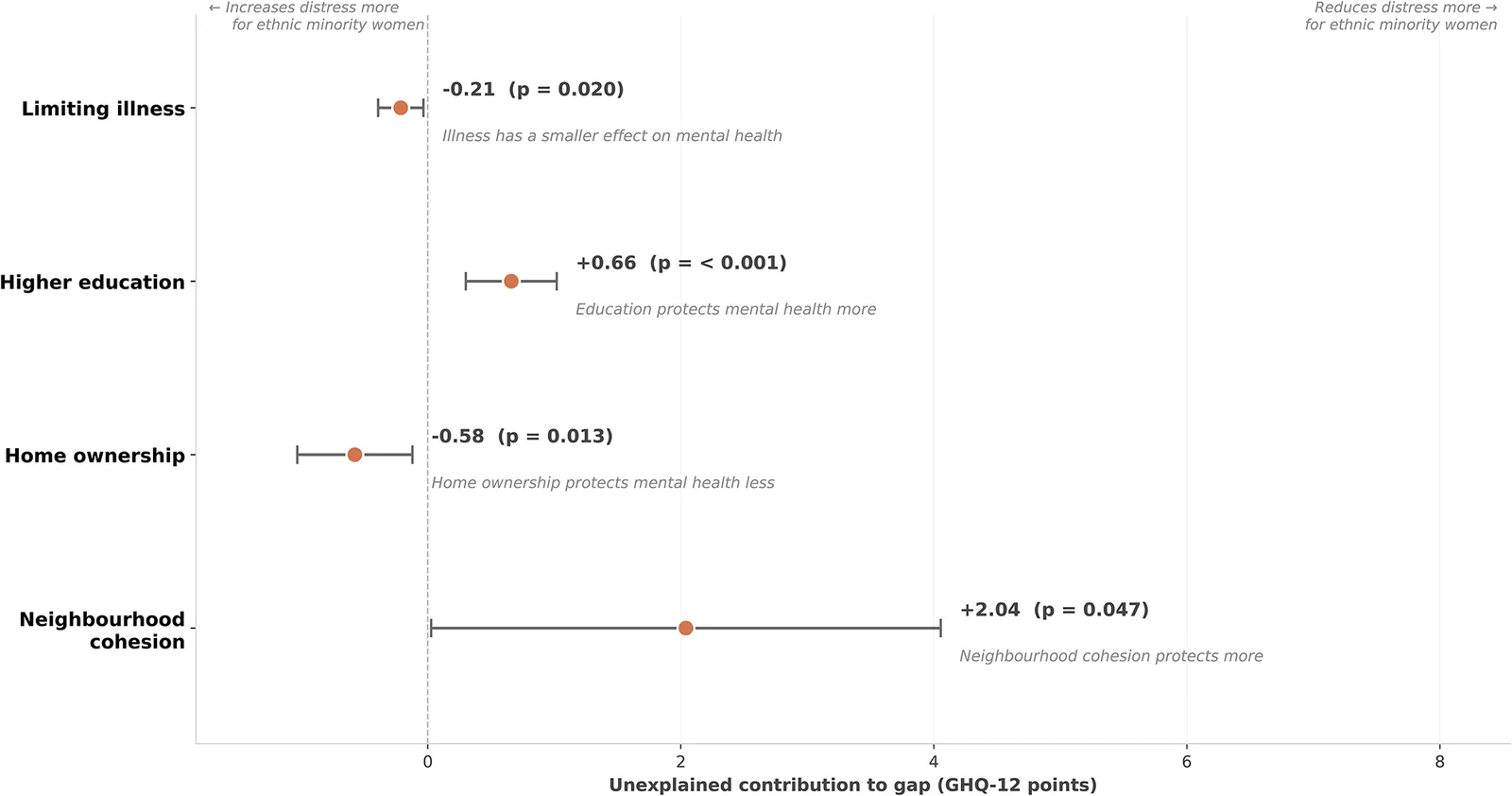
Significant differential vulnerability effects: GHQ-12 psychological distress. Source: UKHLS Waves 2–15 (2010–2024) [33]

The − 0.35 unexplained points, though not significant overall, again concealed important individual effects. Strikingly, the direction of several differential vulnerabilities reversed compared with the SF-12 (Fig. 4).

Education now showed the opposite effect. Holding a higher education qualification was associated with a greater reduction in psychological distress for ethnic minority women (+ 0.66 points, *p* < 0.001). On the SF-12, education protected this group less; on the GHQ, it protected them more.

Neighbourhood cohesion emerged as significant only on the GHQ. Higher cohesion was associated with a substantially greater reduction in distress for ethnic minority women (+ 2.04 points, *p* = 0.047). Home ownership also reversed direction: it was associated with less distress reduction for ethnic minority women (− 0.58 points, *p* = 0.013), whereas on the SF-12 it was more protective. Limiting long-standing illness similarly reversed (− 0.21 points, *p* = 0.020), with illness increasing distress somewhat less for ethnic minority women.

#### Cross-outcome comparison

The reversal of differential vulnerability effects between SF-12 and GHQ is a notable finding. Education protects functional wellbeing (SF-12) less for ethnic minority women but reduces psychological distress (GHQ) more. Home ownership protects functional wellbeing more but reduces distress less. These contrasting patterns likely reflect the different constructs captured by each instrument. The SF-12 mental component captures broader aspects of mental health functioning, including social and role functioning. The GHQ-12 measures recent psychological distress more narrowly. The social determinants examined here may operate differently on these distinct dimensions of mental health.

### Decomposition of the within-women contrast

To address an intracategorical intersectional question, we re-estimated the main decomposition restricting Group 2 to white women only (M6). This intracategorical contrast complements M1: M1 isolates the intersectional gap between ethnic minority women and the rest of the population, while M6 isolates the ethnic gap within women. The two contrasts answer different questions and are reported as co-equal main analyses, following Bauer and Scheim’s [21] discussion of intersectional and stratified comparisons.

#### SF-12 mental health

The raw SF-12 gap between ethnic minority women and white women was 0.60 points and not statistically significant (*p* = 0.13; Table A21). This near-zero raw gap conceals two large, opposing components. The explained component was 1.64 points (*p* < 0.001), meaning the compositional profile of ethnic minority women, including younger age, lower home ownership, lower income, and weaker neighbourhood cohesion, would predict markedly worse functional mental health than white women experience. A negative unexplained component of − 1.05 points (*p* = 0.006) offset this almost entirely. The pattern of explained contributions resembles M1: age and age-squared net to a positive contribution, while home ownership (+ 0.17, *p* = 0.001), neighbourhood cohesion (+ 0.25, *p* < 0.001), and marital status (+ 0.14, *p* = 0.001) each contribute in the expected direction. Because the within-women age contrast is smaller than the intersectional contrast in M1, age accounts for a smaller share of the explained component, and other determinants take more weight. The unexplained component is driven by the same two variables that dominated M1: education returns are less protective for ethnic minority women on the SF-12 (− 1.01, *p* = 0.002), while home ownership returns are more protective (+ 1.21, *p* = 0.006). This pattern is consistent with differential returns favouring ethnic minority women within the female sample, although these returns are themselves shaped by intersectional position and should not be read as resilience.

#### GHQ-12 psychological distress

The within-women GHQ gap was essentially zero (− 0.09 points, *p* = 0.694). The explained component was small but significant (− 0.38, *p* < 0.001), and the unexplained component was non-significant (0.29, *p* = 0.195). Tables A21 and A22 present the full M6 results.

### Sensitivity analyses

We report five sensitivity analyses, ordered as follows: two address the carry-forward imputation strategy (M2 and M3), one addresses within-person dependence (M9), and two address covariate specification (M7 and M8).

#### Reduced model without carry-forward variables

Removing discrimination and neighbourhood cohesion from the decomposition increased the sample to 383,362 observations and widened the SF-12 gap to 2.82 points. Of this larger gap, only 1.46 points (52%) were explained. The remaining 1.36 points (48%) were unexplained and highly significant (*p* < 0.001). The GHQ model showed a parallel shift: the gap widened to − 1.07 points, of which − 0.64 (60%) were unexplained (*p* = 0.001).

The contrast with the main model is clear. In the main model, 85% of the SF-12 gap was explained and the unexplained component was non-significant. Without discrimination and neighbourhood cohesion, the explained share dropped to 52% and the unexplained component became significant. This indicates that these two variables account for a substantial portion of what would otherwise appear as unexplained differential vulnerability. Table A4 and Table A5 present the full results for SF-12 and GHQ, respectively.

#### Observed-only waves without imputation

Restricting the analysis to waves in which discrimination and neighbourhood cohesion were directly observed (*N* = 175,779) yielded a larger SF-12 gap of 3.09 points. The explained component was 1.18 points (38%), and the unexplained component was significant (1.92 points, *p* < 0.001). The GHQ gap was − 1.53 points, with a significant unexplained component (− 1.04, *p* < 0.001).

Within the included waves, both rotating modules are simultaneously observed only in waves 3 and 9, which contributes to the smaller and selectively-waved Group 2 sample (approximately 1,594 ethnic minority women) and to lower precision on individual contributions. M3 is also slightly younger and has a higher ethnic minority women share than M1 (Table A17), but composition alone does not account for the scale of the divergence from M1. Two non-exclusive interpretations remain: the reduced precision of M3, and genuine instability of discrimination and neighbourhood cohesion over the two-year window the carry-forward in M1 spans. We favour the second reading as the more honest one and treat the M1 contribution of these two variables as an upper bound (see Limitations). Tables A6 and A7 present the full M3 results.

#### Within-person clustering

The main analyses cluster standard errors at the primary sampling unit (PSU) level, following the UKHLS svyset specification. To test whether this is adequate given that individuals contribute to multiple waves, we re-estimated M1 with standard errors clustered at the individual level (pidp). Point estimates are unchanged by construction. Standard errors were essentially identical: the explained-component standard error was 0.173 under both clustering schemes, and no covariate changed significance status. Design degrees of freedom rose from 4,799 under PSU clustering to 48,625 under within-person clustering, reflecting the larger number of clusters. We conclude that PSU clustering adequately captures within-person dependence in this dataset, and we retain it for the main analyses. Table A14 and Table A16 present the full M9 results alongside the original M1 standard errors.

#### Continuous log income

As median-splitting household income might produce a counterintuitive income contribution, we tested this by re-estimating M1 with the natural logarithm of equivalised household income in place of the above-median binary indicator (M7). The SF-12 gap was 1.66 points (*p* < 0.001), of which 1.42 (86%) were explained (*p* < 0.001) and 0.24 unexplained (n.s.). The income contribution to the explained component was + 0.043 points (*p* = 0.008), in the expected direction: lower income among ethnic minority women contributed to their worse functional mental health. The overall pattern is consistent with the headline result of M1. The GHQ gap was − 0.71 points (*p* < 0.001), of which − 0.43 (60.6%) were explained, with a significant log-income contribution of − 0.038 points (*p* < 0.001). The explained-versus-unexplained split is robust to the income specification on both outcomes. Tables A23 and A24 present the full M7 results.

#### Removing limiting long-standing illness

To address the concern that limiting long-standing illness lies on the causal pathway between social position and mental health, we re-estimated M1 without that variable on the right-hand side (M8). The SF-12 gap was 1.83 points (*p* < 0.001), of which 1.66 (91%) were explained (*p* < 0.001) and 0.18 unexplained (n.s.). On the GHQ, the gap was − 0.81 points, with − 0.54 (67%) explained and − 0.27 unexplained (n.s.). The decomposition is essentially unchanged from M1, indicating that the over-controlling concern is not load-bearing for the headline result. The near-unchanged explained component is consistent with limiting long-standing illness operating partly as a near-duplicate of age in this population, with much of the variance it carries being absorbed by age and age-squared once illness is removed. Tables A25 and A26 present the full M8 results.

### Secondary analyses

#### Ethnicity without gender

As reported in Section “Mental health gaps across comparison groups”, the raw SF-12 gap between non-white and white respondents was non-significant (0.19 points, *p* = 0.531). The decomposition revealed that this near-zero gap concealed substantial opposing forces. The explained component was + 1.27 points (*p* < 0.001), meaning that the compositional profile of non-white respondents — younger age, lower ownership, higher discrimination — should produce worse mental health. But a significant negative unexplained component (− 1.08, *p* < 0.001) offset this entirely: non-white respondents obtained greater mental health returns from their characteristics than white respondents. The two forces summed to just 0.19 points. On the GHQ, the raw gap was again negligible (0.03, *p* = 0.857), with a small explained component (− 0.23, *p* = 0.004) and a non-significant unexplained component (0.26, *p* = 0.112). Table A8 and Table A9 present the full results for SF-12 and GHQ, respectively.

#### Disaggregated ethnic groups

The disaggregated decompositions revealed distinct patterns for Asian and Black women.

For Asian women, the SF-12 gap of 1.45 points was split between an explained component (0.69 points, *p* = 0.001) and a borderline unexplained component (0.76 points, *p* = 0.082). The GHQ pattern was more striking. The explained component was near zero (− 0.02, *p* = 0.809), and the entire gap of − 0.71 points was attributable to the unexplained component (− 0.69, *p* = 0.004). For Asian women, the psychological distress gap reflected almost entirely differential vulnerability rather than differential exposure.

For Black women, the pattern differed. The SF-12 explained component was large (2.04 points, *p* < 0.001), indicating substantial compositional disadvantage. A significant negative unexplained component (− 1.48, *p* = 0.012) offset this, producing a small, non-significant raw gap (0.56, *p* = 0.423). The GHQ gap was essentially zero (− 0.04, *p* = 0.929), with the explained component (− 0.82, *p* < 0.001) offset by the unexplained component (0.79, *p* = 0.034). Black women experienced considerable compositional disadvantage but this was counterbalanced by differential returns to their characteristics. Table A10 to A13 presents the full disaggregated results for both groups and outcomes.

## Discussion

This study applied the Oaxaca-Blinder decomposition to examine mental health inequities between ethnic minority women and the rest of the UK adult population. The analysis drew on 14 waves of the UK Household Longitudinal Study. Three principal findings emerged. First, a significant mental health gap exists at the intersection of ethnicity and gender that is invisible when ethnicity is examined alone. Second, a substantial share of this gap is associated with differential exposure, particularly age composition, lower home ownership, lower rates of marriage or cohabitation, and weaker neighbourhood cohesion, although the precise share is sensitive to specification. Third, the non-significant overall unexplained component masks substantial and opposing differential vulnerabilities, with the direction of these effects reversing between the two mental health measures.

### Differential exposure and the explained gap

The large explained component on the SF-12 indicates that a substantial share of the mental health gap is associated with differences in observed characteristics, although the partition is sensitive to specification, as we set out in the Sensitivity analyses. It reflects differences in the circumstances ethnic minority women face, not differences in how those circumstances affect health. In the Diderichsen framework, this corresponds to differential exposure: ethnic minority women are disproportionately exposed to conditions that damage mental health [24].

Age composition was the largest contributor to the explained gap, accounting for 1.33 of the 1.55 explained points on the SF-12. This reflects the younger age profile of ethnic minority women and the well-established U-shaped relationship between age and mental health, whereby younger adults report worse mental wellbeing than middle-aged adults. While age is not a modifiable exposure, its large contribution underscores the importance of age-adjusting mental health comparisons between groups with different demographic profiles. It also implies that as the ethnic minority population ages, this component of the gap may narrow naturally.

Beyond age, the explained gap was driven by material and psychosocial disadvantage. Lower rates of home ownership contributed 0.19 points to the SF-12 gap. Home ownership in the UK confers both material security and a sense of control over one’s living environment. Ethnic minority households face well-documented barriers to owner-occupation, including lower wealth accumulation, discrimination in mortgage lending, and concentration in urban rental markets [47].

Lower rates of marriage and cohabitation among ethnic minority women contribute a similar magnitude to the gap. The unmarried category covers never-married, cohabiting, and previously-married statuses with different support patterns, so this contribution should be read as composite rather than as an effect of marriage as such [48].

Lower neighbourhood cohesion contributed 0.16 points. This is consistent with evidence that social cohesion is an important determinant of mental health, one that interacts with deprivation and ethnicity in complex ways [49]. Higher exposure to discrimination added 0.11 points. This aligns with the substantial UK literature linking experiences of racial harassment to adverse mental health outcomes [48, 50, 51].

The sensitivity analysis reinforced the importance of discrimination and neighbourhood cohesion. When these two variables were removed from the model, the explained share of the SF-12 gap fell from 85% to 52%, and the unexplained component became highly significant. This suggests that discrimination and neighbourhood cohesion are not merely additional covariates but key mechanisms through which the mental health gap operates. Without them, a substantial portion of the gap appears as unexplained differential vulnerability — when in fact it reflects measurable and modifiable exposures.

These findings are broadly consistent with Brydsten et al. [27], who found that social integration factors — including financial strain, social relations, and civic engagement — explained large proportions of mental health differences between native-born and foreign-born individuals in Sweden. Our results extend this evidence to an intersectional comparison in the UK context and identify the specific social determinants that matter most. Similarly, Jester et al. [30] found that social determinants of health explained most racial and ethnic disparities in psychological wellbeing among older adults in the United States. Our findings confirm this pattern in a younger, UK-based population using an intersectional grouping. Meulman et al. [29] reached a similar conclusion for socioeconomic differences in self-rated health in the Netherlands, where social determinants accounted for nearly all of the income gap.

Two factors worked in the opposite direction, narrowing the gap. Lower rates of limiting long-standing illness among ethnic minority women (reflecting their younger age) and higher employment participation both conferred relative advantage. These offsetting effects are substantively important: they indicate that ethnic minority women possess characteristics that should, all else being equal, give them better mental health than the rest of the population. The observed gap exists despite these advantages.

### Differential vulnerability: opposing forces beneath the surface

Although the overall unexplained component was non-significant on the SF-12 (0.28 points, *p* = 0.417), this apparent null finding concealed large and statistically significant effects operating in opposite directions. In the Diderichsen framework, the unexplained component contains differential vulnerability, the same social determinant producing different mental health returns by social position. It also contains unmeasured group differences, measurement error, and specification dependence. We treat the framework as a guiding interpretation rather than a definitive empirical partition.

The most striking finding was the reduced protective effect of education. Higher education was associated with 1.19 fewer SF-12 points of benefit for ethnic minority women compared with the rest of the population. This suggests that education does not translate into the same mental health advantages for this group. Several mechanisms may explain this pattern. Labour market evidence consistently shows that ethnic minority graduates in the UK face significant pay penalties even after controlling for qualifications, occupation, and region [52, 53]. A recent Joseph Rowntree Foundation report found that higher qualifications alone do not equalise labour market outcomes for ethnic minority workers, with significant reward gaps persisting particularly among Black and Pakistani/Bangladeshi groups. If education fails to deliver expected returns in terms of employment quality, income, and social status, its mental health benefits may be correspondingly diminished [54]. Additionally, ethnic minority women in higher education and professional settings may face gendered racism — discrimination at the intersection of race and gender. This creates additional psychological burden precisely in the environments where education should confer advantage [19].

Home ownership showed the opposite pattern, conferring a greater mental health benefit for ethnic minority women (+ 1.05 points on the SF-12). This may reflect the particular significance of housing security for a group that faces elevated housing precarity. For ethnic minority women who do achieve owner-occupation, the psychological benefit of that security — stability, autonomy, neighbourhood attachment — may be proportionally greater than for a population where ownership is more normatively expected. This finding suggests that policies facilitating home ownership among ethnic minority households could yield disproportionately large mental health returns.

These opposing effects — education protecting less but ownership protecting more — largely cancelled each other out, producing a non-significant aggregate. This cancellation is methodologically important. It demonstrates that relying solely on the overall unexplained component can obscure meaningful differential vulnerabilities. Researchers using decomposition methods should routinely examine individual variable contributions within the unexplained component, even when the aggregate is non-significant. Several plausibly relevant variables are not in our specification, including cultural and family resources, religious participation, language and acculturation, neighbourhood features beyond cohesion, and mental health service use. We cannot quantify their contribution from the available data. Some of what appears as differential vulnerability may therefore be residual confounding by group-correlated unobservables.

The discrimination measure is asymmetric across groups, conflating racial, religious, and gender motivations in proportions we cannot disaggregate. This affects the interpretation of both its explained-component contribution and any differential return.

### The SF-12/GHQ divergence: two dimensions of mental health

A notable finding was the reversal of differential vulnerability effects between the SF-12 and the GHQ-12. Education protected functional wellbeing (SF-12) less for ethnic minority women but reduced psychological distress (GHQ-12) more. Home ownership protected functional wellbeing more but reduced distress less. Neighbourhood cohesion showed a large differential effect only on the GHQ (+ 2.04 points, *p* = 0.047). This suggests that social cohesion matters considerably more for the acute psychological distress of ethnic minority women than for the rest of the population.

These contrasting patterns likely reflect the different constructs captured by each instrument. The SF-12 Mental Component Summary measures broader functional aspects of mental health, incorporating social and role functioning alongside emotional wellbeing. The GHQ-12 captures recent psychological distress more narrowly — the experience of symptoms such as anxiety, loss of confidence, and inability to concentrate in the past few weeks. These are related but distinct dimensions of mental health, and the social determinants examined here appear to operate differently on each.

The education reversal is particularly instructive. Education may fail to protect the functional dimension of mental health (SF-12) because its expected returns — career progression, social status, financial security — are structurally constrained by labour market discrimination. But it may nevertheless protect against acute psychological distress (GHQ-12) through cognitive resources, health literacy, and coping strategies that buffer day-to-day emotional wellbeing. Similarly, neighbourhood cohesion may operate primarily as a buffer against acute distress, providing immediate social support and a sense of belonging. It is less likely to shape the broader functional dimension that depends on material and structural conditions.

The use of two outcome measures is therefore not merely a robustness check but a substantive analytical choice. Studies relying on a single mental health measure risk drawing incomplete or misleading conclusions about how social determinants operate across different dimensions of mental health.

### Intersectionality and within-group heterogeneity

The ethnicity-only analysis provided striking empirical support for the intersectional approach. The raw mental health gap between all non-white and white respondents was negligible and non-significant on both measures. Yet beneath this null aggregate, the decomposition revealed substantial opposing forces: a significant positive explained component (+ 1.27 points on the SF-12) offset by a significant negative unexplained component (− 1.08 points). Non-white respondents’ compositional disadvantage was entirely counterbalanced by more favourable returns to their characteristics.

This finding validates the intersectional premise articulated by Crenshaw [18]: examining ethnicity in isolation obscures inequities that emerge only when gender is simultaneously considered. The mental health gap becomes visible only at the intersection of minority ethnicity and female gender. Single-axis analyses would conclude that no significant ethnic mental health gap exists in the UK — a finding that would be technically accurate but substantively misleading. Alternative quantitative intersectional methods such as MAIHDA [55] describe intersectional variation but do not decompose the gap into exposure and vulnerability components. The Oaxaca-Blinder approach fills this role. Our analytical strategy combines two complementary intersectional contrasts. M1 is intercategorical: it asks how the joint position of being an ethnic minority woman differs from the position of not being one. M6 is intracategorical: it asks how that joint position differs from the gender-matched comparator [45].

The disaggregated analysis revealed further complexity. For Asian women, the GHQ-12 gap was driven almost entirely by differential vulnerability (unexplained component: −0.69, *p* = 0.004), with virtually no compositional contribution. This points towards structural discrimination and institutional barriers that affect how social determinants translate into psychological distress for this group. Experiences of gendered Islamophobia, cultural expectations around mental health disclosure, and barriers to accessing culturally appropriate services may all contribute [15, 16].

For Black women, the pattern was markedly different. Substantial compositional disadvantage (explained component: 2.04 points on the SF-12) was counterbalanced by protective differential returns (unexplained component: −1.48, *p* = 0.012), producing a small, non-significant raw gap. This resilience — whereby Black women obtain greater mental health returns from their characteristics than the reference population — merits careful interpretation. As noted in the Background, disadvantaged groups may develop coping strategies, community networks, and cultural resources that buffer the effects of structural adversity. Research on ethnic density effects suggests that co-ethnic community concentration may protect against some of the mental health consequences of racial harassment [56]. However, these protective mechanisms should not be used to minimise the severity of the structural disadvantages Black women face. The compositional disadvantage is real and substantial; it is the differential returns that prevent it from manifesting as a raw outcome gap.

### Strengths, limitations, and policy implications

#### This study has several strengths

It draws on approximately 292,000 person-wave observations from 14 waves of a nationally representative survey with an ethnic minority boost sample, providing substantial statistical power. The use of two complementary mental health measures reveals how social determinants operate differently across dimensions of mental health. The intersectional design identifies a gap invisible to single-axis analyses. The systematic comparison of models with and without key psychosocial variables clarifies the mechanisms through which the gap operates. The UKHLS is designed to be representative of the UK adult population, and the ethnic minority boost sample strengthens coverage of non-white groups. The findings are therefore generalisable to the UK population, though transferability to other national contexts requires caution given differences in ethnic composition, healthcare systems, and discrimination patterns.

#### Several limitations should be acknowledged

First, the pooled cross-sectional design does not support causal inference. The decomposition identifies associations between social determinants and mental health gaps but cannot establish that changing these determinants would close the gap. Second, the binary white/non-white classification, while necessary for the primary decomposition, aggregates substantial heterogeneity. The disaggregated analyses partially address this but sample size constraints preclude more granular ethnic group comparisons. Third, carry-forward imputation was used for rotating module variables (discrimination and neighbourhood cohesion), which assumes stability between measurement points. The observed-only sensitivity analysis (M3) diverges materially from the main model on the explained-versus-unexplained partition. Two factors plausibly contribute: the reduced and selectively-waved M3 sample, and the possibility that discrimination and neighbourhood cohesion are not stable enough between rotating-module waves to support carry-forward without bias. We treat the M1 contribution of these two variables as an upper bound. Fourth, cross-sectional survey weights are not available in the protected data file. We use the longitudinal self-completion weight, which calibrates to the longitudinal respondent population including the ethnic minority boost sample. This is the most defensible choice given the data, but it is not equivalent to a true cross-sectional weight at each wave. The boost-sample over-representation may affect group composition in ways that ordinary cross-sectional weights would partly correct. Fifth, health behaviours (smoking, alcohol, diet) were excluded due to insufficient wave coverage, potentially omitting relevant pathways. Sixth, the large contribution of age to the explained component, while genuine, may partly reflect the mechanical effect of comparing groups with very different age distributions rather than a modifiable social determinant. Seventh, pooled (Neumark) coefficients may misattribute differential gradients to the explained component if the age-mental health relationship varies by group. The within-women contrast (M6) reduces but does not eliminate this concern, because pooled coefficients are still used across the two groups it compares. Eighth, both mental health outcomes are self-reported. The SF-12 and the GHQ-12 are validated multi-item instruments with established psychometric properties, and they are more robust than single-item global health ratings. They nonetheless rely on self-completion, as do most of our explanatory variables, including discrimination, neighbourhood cohesion, and limiting long-standing illness. Shared method variance may therefore inflate some associations, since a respondent’s general reporting style can affect both sides of the model. A further concern is specific to decomposition. If reporting thresholds differ systematically by ethnicity or gender, then part of what we observe as differential returns may reflect differences in how groups report similar states. Such measurement differences would load onto the unexplained component and cannot be separated from genuine differential vulnerability. This reinforces our cautious reading of that component throughout.

#### These findings carry several policy implications

The dominance of the explained component suggests that reducing the mental health gap requires improving access to the social determinants that drive it. Specifically, policies should address housing disadvantage among ethnic minority women, including barriers to home ownership and the quality of rental accommodation. The finding that home ownership confers disproportionately large mental health benefits for this group strengthens the case for targeted housing support. Interventions to strengthen neighbourhood cohesion — through community development programmes, investment in local amenities, and support for community organisations — could yield particular benefits for ethnic minority women’s psychological wellbeing. The large differential return identified on the GHQ-12 reinforces this case. Anti-discrimination measures remain a relevant policy lever: the contribution of discrimination exposure to the explained gap is consistent with the view that reducing racial harassment would narrow the mental health inequity, although the observational design does not establish this causally.

The differential vulnerability findings point towards a complementary set of interventions. The reduced protective effect of education on functional mental health suggests that expanding educational access alone is insufficient. Policies would also need to address the labour market discrimination that may prevent ethnic minority women from realising the expected returns to their qualifications. Mandatory ethnicity pay gap reporting, targeted career progression support, and enforcement of anti-discrimination legislation in the workplace are relevant policy levers. Evidence that UK mental health services operate within monocultural frameworks that fail to accommodate ethnic minority experiences is relevant here [15]. Culturally adapted services could help reduce differential vulnerability by ensuring that available resources deliver equivalent mental health benefits regardless of ethnicity and gender.

A multilevel or fixed-effects specification would handle within-person dependence differently, but neither delivers the explained-versus-unexplained partition that maps onto the Diderichsen framework, and a fixed-effects design would discard the between-person variation that is central to an intersectional contrast.

## Conclusions

The mental health gap between ethnic minority women and the rest of the UK population is substantial, and a large share is associated with differences in observed characteristics, notably age composition, home ownership, marital status, and neighbourhood cohesion. The unexplained component contains differential vulnerability in the Diderichsen sense but also unmeasured group differences, measurement error, and specification dependence; we treat it as informative rather than definitive. Significant differential returns operate in opposing directions beneath the surface. The gap is invisible along ethnicity alone and becomes apparent only through an intersectional lens. The within-women contrast confirms that this intersectional gap reflects ethnic inequities operating among women, with compositional disadvantage offset by differential returns favouring ethnic minority women within the female sample. These findings are consistent with two complementary directions for policy: redistributing access to the social determinants associated with much of the observed gap, and addressing the structural conditions under which those determinants translate into mental health returns. The decomposition does not identify which lever is causally larger, and the specification sensitivity reported in the Sensitivity analyses means our findings should be read as motivating rather than ranking these directions.

## Supplementary Information

Below is the link to the electronic supplementary material.

Supplementary Material 1: Additional file 1: Supplementary tables (.docx). Descriptive statistics, sample-construction tables (attrition flow, M3 versus M1 composition, intra-class correlations, carry-forward breakdown, wave-contribution distribution), and full Oaxaca-Blinder decomposition results for all models (Tables A1–A26)

## Data Availability

The data used in this study are available from the UK Data Service (https://ukdataservice.ac.uk/). Access to the UKHLS Special Licence data requires application through the UK Data Service.
