# Supplementary material for: Decomposing mental health inequities: an Oaxaca-Blinder analysis of ethnic and gender disparities in the UK
Source: Int J Equity Health. 2026 Aug 17;25:282. doi: 10.1186/s12939-026-02980-2 (PMC13757230; doi:10.1186/s12939-026-02980-2)
Supplement: Supplementary file 1 [file 12939_2026_2980_MOESM1_ESM.docx]

**Table A1.** Descriptive statistics by ethnic group (M1 SF-12 analysis sample, N=291,711)

| **Descriptive statistics by ethnic group** | | | | | |
| --- | --- | --- | --- | --- | --- |
| **Variable** | **White (N=224,361)** | **Mixed (N=7,453)** | **Asian (N=41,203)** | **Black (N=15,138)** | **Other (N=2,309)** |
| Age (years) | 49.5 (15.4) | 37.4 (9.3) | 39.0 (9.7) | 42.4 (9.7) | 43.4 (9.5) |
| Married or cohabiting | 60.2 | 39.8 | 62.7 | 39.9 | 53.0 |
| Children in household | 28.4 | 35.2 | 52.0 | 48.2 | 48.7 |
| Limiting long-standing illness | 23.4 | 19.4 | 12.6 | 15.5 | 23.0 |
| Experienced discrimination | 21.5 | 21.9 | 22.2 | 21.1 | 18.3 |
| Above median household income | 64.8 | 59.2 | 49.9 | 45.9 | 45.4 |
| Employed or student | 66.7 | 87.9 | 80.2 | 81.7 | 67.3 |
| Higher education | 39.3 | 48.4 | 49.6 | 48.0 | 52.7 |
| Home owner | 71.6 | 56.8 | 72.0 | 34.7 | 47.1 |
| Rural residence | 24.6 | 13.8 | 3.2 | 1.8 | 8.1 |
| Neighbourhood cohesion (1-5) | 3.5 (0.6) | 3.3 (0.5) | 3.5 (0.5) | 3.3 (0.4) | 3.5 (0.4) |
| SF-12 Mental Health (0-100) | 48.3 (8.6) | 45.9 (6.9) | 47.7 (6.8) | 48.3 (6.4) | 46.4 (5.8) |
| GHQ-12 Likert (0-36) | 11.4 (4.5) | 12.3 (4.0) | 11.4 (3.9) | 11.2 (3.8) | 12.5 (3.6) |

*Source: UKHLS waves 2-15 (2010-2024). Survey-weighted estimates. Sample restricted to M1 SF-12 analysis sample (N=291,711). Continuous variables shown as mean (SD); binary variables as %.*

**Table A2.** Oaxaca-Blinder decomposition of SF-12 Mental Component: ethnic minority women vs rest of population

| **Blinder-Oaxaca decomposition** | | | | | | |
| --- | --- | --- | --- | --- | --- | --- |
| Group 1: Rest of the UK population | | | | Number of observations: 291,711 | | |
| Group 2: Ethnic minority women | | | | Model: Linear (pooled) | | |
| **SF-12 Mental Component** | **Coeff.** | **Linearised SE** | **t** | **P>\|t\|** | **[95% Conf.** | **Interval]** |
| **Overall** |  |  |  |  |  |  |
| Group 1 mean | 47.854 | 0.123 | 389.78 | <0.001 | 47.613 | 48.095 |
| Group 2 mean | 46.023 | 0.363 | 126.67 | <0.001 | 45.311 | 46.735 |
| Difference | 1.831 | 0.380 | 4.82 | <0.001 | 1.085 | 2.576 |
| Explained | 1.547 | 0.174 | 8.91 | <0.001 | 1.206 | 1.887 |
| Unexplained | 0.284 | 0.350 | 0.81 | 0.417 | -0.402 | 0.971 |
|  |  |  |  |  |  |  |
| **Explained** |  |  |  |  |  |  |
| Age | -1.520 | 0.280 | -5.43 | <0.001 | -2.068 | -0.971 |
| Age squared | 2.847 | 0.323 | 8.81 | <0.001 | 2.214 | 3.481 |
| Married/cohabiting | 0.198 | 0.042 | 4.68 | <0.001 | 0.115 | 0.281 |
| Children in household | 0.051 | 0.051 | 1.01 | 0.313 | -0.048 | 0.150 |
| Limiting long-standing illness | -0.250 | 0.069 | -3.64 | <0.001 | -0.385 | -0.115 |
| Discrimination exposure | 0.108 | 0.046 | 2.34 | 0.020 | 0.017 | 0.198 |
| Above median income | -0.088 | 0.031 | -2.81 | 0.005 | -0.149 | -0.026 |
| Employed or student | -0.104 | 0.030 | -3.44 | <0.001 | -0.163 | -0.045 |
| Higher education | -0.018 | 0.012 | -1.50 | 0.133 | -0.042 | 0.006 |
| Home ownership | 0.193 | 0.047 | 4.08 | <0.001 | 0.100 | 0.285 |
| Rural residence | -0.034 | 0.044 | -0.78 | 0.434 | -0.120 | 0.052 |
| Neighbourhood cohesion | 0.164 | 0.064 | 2.55 | 0.011 | 0.038 | 0.289 |
|  |  |  |  |  |  |  |
| **Unexplained** |  |  |  |  |  |  |
| Age | 8.351 | 4.338 | 1.93 | 0.054 | -0.153 | 16.856 |
| Age squared | -5.210 | 2.295 | -2.27 | 0.023 | -9.710 | -0.711 |
| Married/cohabiting | -0.360 | 0.358 | -1.01 | 0.315 | -1.062 | 0.342 |
| Children in household | -0.598 | 0.311 | -1.92 | 0.055 | -1.208 | 0.012 |
| Limiting long-standing illness | 0.280 | 0.142 | 1.97 | 0.049 | 0.002 | 0.558 |
| Discrimination exposure | -0.082 | 0.159 | -0.52 | 0.606 | -0.393 | 0.229 |
| Above median income | 0.512 | 0.285 | 1.80 | 0.072 | -0.045 | 1.070 |
| Employed or student | 0.193 | 0.559 | 0.35 | 0.730 | -0.903 | 1.289 |
| Higher education | -1.193 | 0.320 | -3.73 | <0.001 | -1.819 | -0.566 |
| Home ownership | 1.052 | 0.408 | 2.58 | 0.010 | 0.253 | 1.851 |
| Rural residence | 0.093 | 0.092 | 1.01 | 0.314 | -0.088 | 0.273 |
| Neighbourhood cohesion | -1.899 | 1.653 | -1.15 | 0.251 | -5.141 | 1.342 |
| Constant | -0.855 | 2.595 | -0.33 | 0.742 | -5.941 | 4.232 |

*Married/cohabiting: Legally married or cohabiting with partner*

*Children in household: One or more children aged under 16 in household*

*Limiting long-standing illness: Long-standing health condition that limits daily activities*

*Discrimination exposure: Physically attacked or avoided places due to fear of attack based on ethnicity, religion, or other characteristic*

*Above median income: Equivalised household income above survey-weighted population median*

*Employed or student: Currently in employment or full-time education*

*Higher education: Degree-level qualification or other higher education*

*Home ownership: Owns home outright or with mortgage*

*Rural residence: Rural area (ONS classification)*

*Neighbourhood cohesion: Scale of neighbourhood belonging and trust, 1-5 (carry-forward imputed from rotating module waves)*

*Source: UK Household Longitudinal Study (UKHLS), waves 2-15 (2010-2024). Survey-weighted Oaxaca-Blinder decomposition with pooled coefficients. Linearised standard errors account for complex survey design.*

**Table A3.** Oaxaca-Blinder decomposition of GHQ-12 Likert: ethnic minority women vs rest of population

| **Blinder-Oaxaca decomposition** | | | | | | |
| --- | --- | --- | --- | --- | --- | --- |
| Group 1: Rest of the UK population | | | | Number of observations: 292,586 | | |
| Group 2: Ethnic minority women | | | | Model: Linear (pooled) | | |
| **GHQ-12 Likert** | **Coeff.** | **Linearised SE** | **t** | **P>\|t\|** | **[95% Conf.** | **Interval]** |
| **Overall** |  |  |  |  |  |  |
| Group 1 mean | 11.536 | 0.061 | 190.43 | <0.001 | 11.417 | 11.655 |
| Group 2 mean | 12.348 | 0.212 | 58.23 | <0.001 | 11.932 | 12.763 |
| Difference | -0.811 | 0.217 | -3.74 | <0.001 | -1.237 | -0.386 |
| Explained | -0.466 | 0.088 | -5.28 | <0.001 | -0.639 | -0.293 |
| Unexplained | -0.346 | 0.201 | -1.72 | 0.085 | -0.739 | 0.048 |
|  |  |  |  |  |  |  |
| **Explained** |  |  |  |  |  |  |
| Age | 1.367 | 0.164 | 8.34 | <0.001 | 1.045 | 1.688 |
| Age squared | -1.783 | 0.178 | -10.03 | <0.001 | -2.132 | -1.434 |
| Married/cohabiting | -0.077 | 0.019 | -4.00 | <0.001 | -0.114 | -0.039 |
| Children in household | -0.030 | 0.027 | -1.11 | 0.267 | -0.084 | 0.023 |
| Limiting long-standing illness | 0.165 | 0.044 | 3.72 | <0.001 | 0.078 | 0.252 |
| Discrimination exposure | -0.049 | 0.022 | -2.25 | 0.025 | -0.092 | -0.006 |
| Above median income | 0.006 | 0.017 | 0.35 | 0.725 | -0.027 | 0.039 |
| Employed or student | 0.058 | 0.017 | 3.41 | <0.001 | 0.025 | 0.091 |
| Higher education | 0.007 | 0.005 | 1.25 | 0.212 | -0.004 | 0.018 |
| Home ownership | -0.090 | 0.024 | -3.81 | <0.001 | -0.137 | -0.044 |
| Rural residence | 0.047 | 0.023 | 2.04 | 0.041 | 0.002 | 0.091 |
| Neighbourhood cohesion | -0.085 | 0.034 | -2.54 | 0.011 | -0.151 | -0.019 |
|  |  |  |  |  |  |  |
| **Unexplained** |  |  |  |  |  |  |
| Age | -2.476 | 2.512 | -0.99 | 0.324 | -7.401 | 2.449 |
| Age squared | 1.591 | 1.291 | 1.23 | 0.218 | -0.939 | 4.122 |
| Married/cohabiting | 0.287 | 0.214 | 1.34 | 0.180 | -0.133 | 0.706 |
| Children in household | 0.116 | 0.179 | 0.65 | 0.516 | -0.234 | 0.466 |
| Limiting long-standing illness | -0.213 | 0.091 | -2.34 | 0.020 | -0.392 | -0.034 |
| Discrimination exposure | -0.042 | 0.092 | -0.46 | 0.649 | -0.223 | 0.139 |
| Above median income | -0.243 | 0.166 | -1.47 | 0.142 | -0.568 | 0.082 |
| Employed or student | -0.068 | 0.300 | -0.23 | 0.820 | -0.657 | 0.520 |
| Higher education | 0.660 | 0.183 | 3.60 | <0.001 | 0.301 | 1.019 |
| Home ownership | -0.576 | 0.232 | -2.48 | 0.013 | -1.031 | -0.121 |
| Rural residence | -0.085 | 0.057 | -1.48 | 0.138 | -0.197 | 0.027 |
| Neighbourhood cohesion | 2.041 | 1.027 | 1.99 | 0.047 | 0.027 | 4.055 |
| Constant | -1.337 | 1.544 | -0.87 | 0.387 | -4.364 | 1.691 |

*Married/cohabiting: Legally married or cohabiting with partner*

*Children in household: One or more children aged under 16 in household*

*Limiting long-standing illness: Long-standing health condition that limits daily activities*

*Discrimination exposure: Physically attacked or avoided places due to fear of attack based on ethnicity, religion, or other characteristic*

*Above median income: Equivalised household income above survey-weighted population median*

*Employed or student: Currently in employment or full-time education*

*Higher education: Degree-level qualification or other higher education*

*Home ownership: Owns home outright or with mortgage*

*Rural residence: Rural area (ONS classification)*

*Neighbourhood cohesion: Scale of neighbourhood belonging and trust, 1-5 (carry-forward imputed from rotating module waves)*

*Source: UK Household Longitudinal Study (UKHLS), waves 2-15 (2010-2024). Survey-weighted Oaxaca-Blinder decomposition with pooled coefficients. Linearised standard errors account for complex survey design.*

**Table A4.** Oaxaca-Blinder decomposition of SF-12 Mental Component: ethnic minority women vs rest (without discrimination and neighbourhood cohesion)

| **Blinder-Oaxaca decomposition** | | | | | | |
| --- | --- | --- | --- | --- | --- | --- |
| Group 1: Rest of the UK population | | | | Number of observations: 383,362 | | |
| Group 2: Ethnic minority women | | | | Model: Linear (pooled) | | |
| **SF-12 Mental Component** | **Coeff.** | **Linearised SE** | **t** | **P>\|t\|** | **[95% Conf.** | **Interval]** |
| **Overall** |  |  |  |  |  |  |
| Group 1 mean | 48.899 | 0.074 | 657.80 | <0.001 | 48.753 | 49.045 |
| Group 2 mean | 46.082 | 0.331 | 139.30 | <0.001 | 45.434 | 46.731 |
| Difference | 2.817 | 0.338 | 8.32 | <0.001 | 2.153 | 3.480 |
| Explained | 1.459 | 0.109 | 13.42 | <0.001 | 1.246 | 1.672 |
| Unexplained | 1.358 | 0.325 | 4.17 | <0.001 | 0.720 | 1.996 |
|  |  |  |  |  |  |  |
| **Explained** |  |  |  |  |  |  |
| Age | -1.298 | 0.173 | -7.52 | <0.001 | -1.637 | -0.960 |
| Age squared | 2.568 | 0.220 | 11.68 | <0.001 | 2.137 | 3.000 |
| Married/cohabiting | 0.212 | 0.035 | 6.03 | <0.001 | 0.143 | 0.281 |
| Children in household | 0.035 | 0.031 | 1.10 | 0.271 | -0.027 | 0.096 |
| Limiting long-standing illness | -0.267 | 0.060 | -4.48 | <0.001 | -0.383 | -0.150 |
| Above median income | -0.007 | 0.007 | -1.07 | 0.284 | -0.021 | 0.006 |
| Employed or student | -0.122 | 0.026 | -4.69 | <0.001 | -0.173 | -0.071 |
| Higher education | 0.002 | 0.014 | 0.12 | 0.906 | -0.026 | 0.029 |
| Home ownership | 0.229 | 0.039 | 5.88 | <0.001 | 0.153 | 0.305 |
| Rural residence | 0.107 | 0.029 | 3.70 | <0.001 | 0.050 | 0.164 |
|  |  |  |  |  |  |  |
| **Unexplained** |  |  |  |  |  |  |
| Age | 8.720 | 3.956 | 2.20 | 0.028 | 0.965 | 16.475 |
| Age squared | -5.653 | 2.109 | -2.68 | 0.007 | -9.787 | -1.519 |
| Married/cohabiting | -0.538 | 0.320 | -1.68 | 0.093 | -1.165 | 0.089 |
| Children in household | -0.824 | 0.302 | -2.73 | 0.006 | -1.417 | -0.232 |
| Limiting long-standing illness | 0.341 | 0.127 | 2.68 | 0.007 | 0.091 | 0.590 |
| Above median income | 0.746 | 0.265 | 2.82 | 0.005 | 0.227 | 1.265 |
| Employed or student | 0.571 | 0.501 | 1.14 | 0.254 | -0.411 | 1.552 |
| Higher education | -1.131 | 0.296 | -3.82 | <0.001 | -1.711 | -0.551 |
| Home ownership | 0.888 | 0.361 | 2.46 | 0.014 | 0.181 | 1.595 |
| Rural residence | 0.103 | 0.076 | 1.36 | 0.173 | -0.045 | 0.252 |
| Constant | -1.865 | 1.824 | -1.02 | 0.307 | -5.440 | 1.711 |

*Married/cohabiting: Legally married or cohabiting with partner*

*Children in household: One or more children aged under 16 in household*

*Limiting long-standing illness: Long-standing health condition that limits daily activities*

*Discrimination exposure: Physically attacked or avoided places due to fear of attack based on ethnicity, religion, or other characteristic*

*Above median income: Equivalised household income above survey-weighted population median*

*Employed or student: Currently in employment or full-time education*

*Higher education: Degree-level qualification or other higher education*

*Home ownership: Owns home outright or with mortgage*

*Rural residence: Rural area (ONS classification)*

*Neighbourhood cohesion: Scale of neighbourhood belonging and trust, 1-5 (carry-forward imputed from rotating module waves)*

*Source: UK Household Longitudinal Study (UKHLS), waves 2-15 (2010-2024). Survey-weighted Oaxaca-Blinder decomposition with pooled coefficients. Linearised standard errors account for complex survey design.*

**Table A5.** Oaxaca-Blinder decomposition of GHQ-12 Likert: ethnic minority women vs rest (without discrimination and neighbourhood cohesion)

| **Blinder-Oaxaca decomposition** | | | | | | |
| --- | --- | --- | --- | --- | --- | --- |
| Group 1: Rest of the UK population | | | | Number of observations: 384,765 | | |
| Group 2: Ethnic minority women | | | | Model: Linear (pooled) | | |
| **GHQ-12 Likert** | **Coeff.** | **Linearised SE** | **t** | **P>\|t\|** | **[95% Conf.** | **Interval]** |
| **Overall** |  |  |  |  |  |  |
| Group 1 mean | 11.186 | 0.037 | 306.41 | <0.001 | 11.114 | 11.257 |
| Group 2 mean | 12.255 | 0.193 | 63.65 | <0.001 | 11.878 | 12.633 |
| Difference | -1.070 | 0.195 | -5.50 | <0.001 | -1.451 | -0.688 |
| Explained | -0.429 | 0.058 | -7.38 | <0.001 | -0.543 | -0.315 |
| Unexplained | -0.640 | 0.187 | -3.43 | <0.001 | -1.007 | -0.274 |
|  |  |  |  |  |  |  |
| **Explained** |  |  |  |  |  |  |
| Age | 1.168 | 0.109 | 10.75 | <0.001 | 0.955 | 1.381 |
| Age squared | -1.633 | 0.126 | -12.91 | <0.001 | -1.880 | -1.385 |
| Married/cohabiting | -0.078 | 0.014 | -5.42 | <0.001 | -0.107 | -0.050 |
| Children in household | -0.012 | 0.017 | -0.71 | 0.479 | -0.045 | 0.021 |
| Limiting long-standing illness | 0.186 | 0.040 | 4.60 | <0.001 | 0.107 | 0.266 |
| Above median income | -0.007 | 0.004 | -1.95 | 0.051 | -0.015 | 0.000 |
| Employed or student | 0.068 | 0.014 | 4.71 | <0.001 | 0.039 | 0.096 |
| Higher education | 0.004 | 0.007 | 0.52 | 0.601 | -0.010 | 0.018 |
| Home ownership | -0.100 | 0.018 | -5.62 | <0.001 | -0.134 | -0.065 |
| Rural residence | -0.025 | 0.015 | -1.67 | 0.095 | -0.053 | 0.004 |
|  |  |  |  |  |  |  |
| **Unexplained** |  |  |  |  |  |  |
| Age | -2.638 | 2.201 | -1.20 | 0.231 | -6.953 | 1.677 |
| Age squared | 1.876 | 1.124 | 1.67 | 0.095 | -0.328 | 4.080 |
| Married/cohabiting | 0.355 | 0.192 | 1.85 | 0.064 | -0.021 | 0.732 |
| Children in household | 0.269 | 0.174 | 1.55 | 0.122 | -0.072 | 0.611 |
| Limiting long-standing illness | -0.214 | 0.079 | -2.72 | 0.007 | -0.368 | -0.060 |
| Above median income | -0.382 | 0.157 | -2.44 | 0.015 | -0.688 | -0.075 |
| Employed or student | -0.260 | 0.249 | -1.05 | 0.296 | -0.748 | 0.228 |
| Higher education | 0.555 | 0.166 | 3.34 | <0.001 | 0.230 | 0.881 |
| Home ownership | -0.407 | 0.209 | -1.95 | 0.052 | -0.818 | 0.003 |
| Rural residence | -0.083 | 0.047 | -1.76 | 0.079 | -0.175 | 0.009 |
| Constant | 0.287 | 1.066 | 0.27 | 0.787 | -1.801 | 2.376 |

*Married/cohabiting: Legally married or cohabiting with partner*

*Children in household: One or more children aged under 16 in household*

*Limiting long-standing illness: Long-standing health condition that limits daily activities*

*Discrimination exposure: Physically attacked or avoided places due to fear of attack based on ethnicity, religion, or other characteristic*

*Above median income: Equivalised household income above survey-weighted population median*

*Employed or student: Currently in employment or full-time education*

*Higher education: Degree-level qualification or other higher education*

*Home ownership: Owns home outright or with mortgage*

*Rural residence: Rural area (ONS classification)*

*Neighbourhood cohesion: Scale of neighbourhood belonging and trust, 1-5 (carry-forward imputed from rotating module waves)*

*Source: UK Household Longitudinal Study (UKHLS), waves 2-15 (2010-2024). Survey-weighted Oaxaca-Blinder decomposition with pooled coefficients. Linearised standard errors account for complex survey design.*

**Table A6.** Oaxaca-Blinder decomposition of SF-12 Mental Component: ethnic minority women vs rest (observed-only waves)

| **Blinder-Oaxaca decomposition** | | | | | | |
| --- | --- | --- | --- | --- | --- | --- |
| Group 1: Rest of the UK population | | | | Number of observations: 175,779 | | |
| Group 2: Ethnic minority women | | | | Model: Linear (pooled) | | |
| **SF-12 Mental Component** | **Coeff.** | **Linearised SE** | **t** | **P>\|t\|** | **[95% Conf.** | **Interval]** |
| **Overall** |  |  |  |  |  |  |
| Group 1 mean | 49.509 | 0.245 | 201.92 | <0.001 | 49.028 | 49.990 |
| Group 2 mean | 46.414 | 0.448 | 103.52 | <0.001 | 45.535 | 47.293 |
| Difference | 3.095 | 0.499 | 6.21 | <0.001 | 2.117 | 4.072 |
| Explained | 1.179 | 0.220 | 5.36 | <0.001 | 0.748 | 1.611 |
| Unexplained | 1.915 | 0.491 | 3.90 | <0.001 | 0.953 | 2.877 |
|  |  |  |  |  |  |  |
| **Explained** |  |  |  |  |  |  |
| Age | -1.064 | 0.349 | -3.05 | 0.002 | -1.747 | -0.380 |
| Age squared | 1.554 | 0.392 | 3.96 | <0.001 | 0.785 | 2.323 |
| Married/cohabiting | 0.143 | 0.069 | 2.07 | 0.038 | 0.008 | 0.277 |
| Children in household | -0.005 | 0.067 | -0.07 | 0.945 | -0.136 | 0.127 |
| Limiting long-standing illness | 0.028 | 0.102 | 0.27 | 0.784 | -0.172 | 0.228 |
| Discrimination exposure | 0.220 | 0.066 | 3.32 | <0.001 | 0.090 | 0.350 |
| Above median income | 0.038 | 0.044 | 0.88 | 0.380 | -0.047 | 0.124 |
| Employed or student | 0.107 | 0.053 | 2.03 | 0.042 | 0.004 | 0.210 |
| Higher education | 0.017 | 0.019 | 0.93 | 0.353 | -0.019 | 0.054 |
| Home ownership | 0.013 | 0.048 | 0.27 | 0.790 | -0.082 | 0.108 |
| Rural residence | 0.025 | 0.072 | 0.35 | 0.727 | -0.116 | 0.167 |
| Neighbourhood cohesion | 0.102 | 0.080 | 1.27 | 0.204 | -0.055 | 0.259 |
|  |  |  |  |  |  |  |
| **Unexplained** |  |  |  |  |  |  |
| Age | 3.903 | 6.248 | 0.62 | 0.532 | -8.347 | 16.153 |
| Age squared | -3.338 | 3.213 | -1.04 | 0.299 | -9.637 | 2.962 |
| Married/cohabiting | -0.369 | 0.610 | -0.61 | 0.545 | -1.565 | 0.826 |
| Children in household | -0.886 | 0.491 | -1.81 | 0.071 | -1.849 | 0.076 |
| Limiting long-standing illness | 0.710 | 0.224 | 3.17 | 0.002 | 0.271 | 1.149 |
| Discrimination exposure | -0.066 | 0.224 | -0.30 | 0.767 | -0.505 | 0.372 |
| Above median income | -0.099 | 0.396 | -0.25 | 0.803 | -0.876 | 0.678 |
| Employed or student | 1.603 | 0.993 | 1.61 | 0.107 | -0.345 | 3.551 |
| Higher education | -0.599 | 0.435 | -1.38 | 0.168 | -1.452 | 0.253 |
| Home ownership | 0.908 | 0.557 | 1.63 | 0.103 | -0.184 | 2.000 |
| Rural residence | 0.151 | 0.094 | 1.60 | 0.109 | -0.034 | 0.335 |
| Neighbourhood cohesion | -3.046 | 2.736 | -1.11 | 0.266 | -8.410 | 2.318 |
| Constant | 3.044 | 3.975 | 0.77 | 0.444 | -4.750 | 10.838 |

*Married/cohabiting: Legally married or cohabiting with partner*

*Children in household: One or more children aged under 16 in household*

*Limiting long-standing illness: Long-standing health condition that limits daily activities*

*Discrimination exposure: Physically attacked or avoided places due to fear of attack based on ethnicity, religion, or other characteristic*

*Above median income: Equivalised household income above survey-weighted population median*

*Employed or student: Currently in employment or full-time education*

*Higher education: Degree-level qualification or other higher education*

*Home ownership: Owns home outright or with mortgage*

*Rural residence: Rural area (ONS classification)*

*Neighbourhood cohesion: Scale of neighbourhood belonging and trust, 1-5 (carry-forward imputed from rotating module waves)*

*Source: UK Household Longitudinal Study (UKHLS), waves 2-15 (2010-2024). Survey-weighted Oaxaca-Blinder decomposition with pooled coefficients. Linearised standard errors account for complex survey design.*

**Table A7.** Oaxaca-Blinder decomposition of GHQ-12 Likert: ethnic minority women vs rest (observed-only waves)

| **Blinder-Oaxaca decomposition** | | | | | | |
| --- | --- | --- | --- | --- | --- | --- |
| Group 1: Rest of the UK population | | | | Number of observations: 175,749 | | |
| Group 2: Ethnic minority women | | | | Model: Linear (pooled) | | |
| **GHQ-12 Likert** | **Coeff.** | **Linearised SE** | **t** | **P>\|t\|** | **[95% Conf.** | **Interval]** |
| **Overall** |  |  |  |  |  |  |
| Group 1 mean | 10.821 | 0.132 | 82.11 | <0.001 | 10.563 | 11.080 |
| Group 2 mean | 12.352 | 0.255 | 48.48 | <0.001 | 11.853 | 12.852 |
| Difference | -1.531 | 0.276 | -5.55 | <0.001 | -2.071 | -0.991 |
| Explained | -0.494 | 0.129 | -3.83 | <0.001 | -0.746 | -0.241 |
| Unexplained | -1.037 | 0.265 | -3.91 | <0.001 | -1.557 | -0.517 |
|  |  |  |  |  |  |  |
| **Explained** |  |  |  |  |  |  |
| Age | 0.856 | 0.213 | 4.02 | <0.001 | 0.439 | 1.273 |
| Age squared | -0.997 | 0.233 | -4.28 | <0.001 | -1.453 | -0.540 |
| Married/cohabiting | -0.055 | 0.037 | -1.48 | 0.138 | -0.127 | 0.018 |
| Children in household | 0.000 | 0.037 | 0.00 | 0.998 | -0.073 | 0.073 |
| Limiting long-standing illness | -0.022 | 0.070 | -0.32 | 0.750 | -0.159 | 0.115 |
| Discrimination exposure | -0.099 | 0.032 | -3.05 | 0.002 | -0.162 | -0.035 |
| Above median income | -0.036 | 0.026 | -1.37 | 0.171 | -0.088 | 0.016 |
| Employed or student | -0.076 | 0.034 | -2.22 | 0.026 | -0.143 | -0.009 |
| Higher education | -0.012 | 0.012 | -1.01 | 0.311 | -0.035 | 0.011 |
| Home ownership | -0.007 | 0.030 | -0.25 | 0.806 | -0.066 | 0.051 |
| Rural residence | 0.019 | 0.037 | 0.50 | 0.618 | -0.055 | 0.092 |
| Neighbourhood cohesion | -0.064 | 0.048 | -1.34 | 0.179 | -0.158 | 0.030 |
|  |  |  |  |  |  |  |
| **Unexplained** |  |  |  |  |  |  |
| Age | 0.280 | 3.701 | 0.08 | 0.940 | -6.976 | 7.536 |
| Age squared | 0.545 | 1.934 | 0.28 | 0.778 | -3.248 | 4.337 |
| Married/cohabiting | -0.006 | 0.363 | -0.02 | 0.987 | -0.718 | 0.706 |
| Children in household | 0.526 | 0.270 | 1.95 | 0.051 | -0.003 | 1.055 |
| Limiting long-standing illness | -0.393 | 0.151 | -2.60 | 0.009 | -0.689 | -0.096 |
| Discrimination exposure | -0.112 | 0.133 | -0.84 | 0.399 | -0.372 | 0.148 |
| Above median income | 0.137 | 0.232 | 0.59 | 0.555 | -0.318 | 0.593 |
| Employed or student | -0.598 | 0.583 | -1.03 | 0.305 | -1.741 | 0.545 |
| Higher education | 0.413 | 0.248 | 1.67 | 0.095 | -0.072 | 0.898 |
| Home ownership | -0.313 | 0.342 | -0.91 | 0.360 | -0.985 | 0.358 |
| Rural residence | -0.090 | 0.062 | -1.45 | 0.147 | -0.213 | 0.032 |
| Neighbourhood cohesion | 2.238 | 1.523 | 1.47 | 0.142 | -0.749 | 5.224 |
| Constant | -3.665 | 2.359 | -1.55 | 0.120 | -8.289 | 0.959 |

*Married/cohabiting: Legally married or cohabiting with partner*

*Children in household: One or more children aged under 16 in household*

*Limiting long-standing illness: Long-standing health condition that limits daily activities*

*Discrimination exposure: Physically attacked or avoided places due to fear of attack based on ethnicity, religion, or other characteristic*

*Above median income: Equivalised household income above survey-weighted population median*

*Employed or student: Currently in employment or full-time education*

*Higher education: Degree-level qualification or other higher education*

*Home ownership: Owns home outright or with mortgage*

*Rural residence: Rural area (ONS classification)*

*Neighbourhood cohesion: Scale of neighbourhood belonging and trust, 1-5 (carry-forward imputed from rotating module waves)*

*Source: UK Household Longitudinal Study (UKHLS), waves 2-15 (2010-2024). Survey-weighted Oaxaca-Blinder decomposition with pooled coefficients. Linearised standard errors account for complex survey design.*

**Table A8.** Oaxaca-Blinder decomposition of SF-12 Mental Component: non-white vs white

| **Blinder-Oaxaca decomposition** | | | | | | |
| --- | --- | --- | --- | --- | --- | --- |
| Group 1: White | | | | Number of observations: 300,321 | | |
| Group 2: Non-white | | | | Model: Linear (pooled) | | |
| **SF-12 Mental Component** | **Coeff.** | **Linearised SE** | **t** | **P>\|t\|** | **[95% Conf.** | **Interval]** |
| **Overall** |  |  |  |  |  |  |
| Group 1 mean | 47.676 | 0.130 | 365.36 | <0.001 | 47.420 | 47.932 |
| Group 2 mean | 47.491 | 0.266 | 178.50 | <0.001 | 46.969 | 48.012 |
| Difference | 0.185 | 0.296 | 0.63 | 0.531 | -0.394 | 0.765 |
| Explained | 1.267 | 0.156 | 8.12 | <0.001 | 0.961 | 1.573 |
| Unexplained | -1.082 | 0.287 | -3.77 | <0.001 | -1.644 | -0.519 |
|  |  |  |  |  |  |  |
| **Explained** |  |  |  |  |  |  |
| Female | 0.023 | 0.031 | 0.73 | 0.466 | -0.038 | 0.083 |
| Age | -1.614 | 0.305 | -5.30 | <0.001 | -2.211 | -1.017 |
| Age squared | 3.113 | 0.337 | 9.23 | <0.001 | 2.452 | 3.774 |
| Married/cohabiting | 0.119 | 0.032 | 3.72 | <0.001 | 0.056 | 0.182 |
| Children in household | 0.040 | 0.049 | 0.82 | 0.413 | -0.056 | 0.137 |
| Limiting long-standing illness | -0.458 | 0.055 | -8.37 | <0.001 | -0.565 | -0.351 |
| Discrimination exposure | 0.005 | 0.035 | 0.14 | 0.885 | -0.064 | 0.074 |
| Above median income | -0.060 | 0.034 | -1.80 | 0.072 | -0.126 | 0.005 |
| Employed or student | -0.190 | 0.046 | -4.13 | <0.001 | -0.280 | -0.100 |
| Higher education | -0.029 | 0.018 | -1.64 | 0.101 | -0.064 | 0.006 |
| Home ownership | 0.199 | 0.046 | 4.28 | <0.001 | 0.108 | 0.290 |
| Rural residence | 0.017 | 0.049 | 0.35 | 0.728 | -0.079 | 0.114 |
| Neighbourhood cohesion | 0.102 | 0.052 | 1.98 | 0.048 | 0.001 | 0.204 |
|  |  |  |  |  |  |  |
| **Unexplained** |  |  |  |  |  |  |
| Female | 0.165 | 0.252 | 0.65 | 0.513 | -0.330 | 0.660 |
| Age | 8.927 | 3.552 | 2.51 | 0.012 | 1.964 | 15.891 |
| Age squared | -4.183 | 1.901 | -2.20 | 0.028 | -7.910 | -0.457 |
| Married/cohabiting | 0.299 | 0.344 | 0.87 | 0.384 | -0.375 | 0.974 |
| Children in household | -0.391 | 0.222 | -1.76 | 0.078 | -0.826 | 0.043 |
| Limiting long-standing illness | 0.189 | 0.110 | 1.72 | 0.086 | -0.027 | 0.404 |
| Discrimination exposure | -0.149 | 0.121 | -1.23 | 0.221 | -0.387 | 0.089 |
| Above median income | -0.017 | 0.231 | -0.07 | 0.941 | -0.470 | 0.436 |
| Employed or student | 0.176 | 0.545 | 0.32 | 0.747 | -0.892 | 1.244 |
| Higher education | -0.786 | 0.245 | -3.20 | 0.001 | -1.266 | -0.305 |
| Home ownership | 1.031 | 0.374 | 2.75 | 0.006 | 0.297 | 1.764 |
| Rural residence | -0.063 | 0.081 | -0.79 | 0.432 | -0.221 | 0.095 |
| Neighbourhood cohesion | -1.414 | 1.216 | -1.16 | 0.245 | -3.798 | 0.969 |
| Constant | -4.865 | 1.893 | -2.57 | 0.010 | -8.575 | -1.154 |

*Married/cohabiting: Legally married or cohabiting with partner*

*Children in household: One or more children aged under 16 in household*

*Limiting long-standing illness: Long-standing health condition that limits daily activities*

*Discrimination exposure: Physically attacked or avoided places due to fear of attack based on ethnicity, religion, or other characteristic*

*Above median income: Equivalised household income above survey-weighted population median*

*Employed or student: Currently in employment or full-time education*

*Higher education: Degree-level qualification or other higher education*

*Home ownership: Owns home outright or with mortgage*

*Rural residence: Rural area (ONS classification)*

*Neighbourhood cohesion: Scale of neighbourhood belonging and trust, 1-5 (carry-forward imputed from rotating module waves)*

*Source: UK Household Longitudinal Study (UKHLS), waves 2-15 (2010-2024). Survey-weighted Oaxaca-Blinder decomposition with pooled coefficients. Linearised standard errors account for complex survey design.*

**Table A9.** Oaxaca-Blinder decomposition of GHQ-12 Likert: non-white vs white

| **Blinder-Oaxaca decomposition** | | | | | | |
| --- | --- | --- | --- | --- | --- | --- |
| Group 1: White | | | | Number of observations: 301,502 | | |
| Group 2: Non-white | | | | Model: Linear (pooled) | | |
| **GHQ-12 Likert** | **Coeff.** | **Linearised SE** | **t** | **P>\|t\|** | **[95% Conf.** | **Interval]** |
| **Overall** |  |  |  |  |  |  |
| Group 1 mean | 11.641 | 0.063 | 184.29 | <0.001 | 11.517 | 11.765 |
| Group 2 mean | 11.611 | 0.157 | 73.87 | <0.001 | 11.302 | 11.919 |
| Difference | 0.031 | 0.169 | 0.18 | 0.857 | -0.301 | 0.363 |
| Explained | -0.227 | 0.078 | -2.90 | 0.004 | -0.380 | -0.074 |
| Unexplained | 0.258 | 0.162 | 1.59 | 0.112 | -0.060 | 0.575 |
|  |  |  |  |  |  |  |
| **Explained** |  |  |  |  |  |  |
| Female | -0.009 | 0.017 | -0.54 | 0.588 | -0.042 | 0.024 |
| Age | 1.505 | 0.171 | 8.79 | <0.001 | 1.169 | 1.841 |
| Age squared | -1.967 | 0.181 | -10.84 | <0.001 | -2.323 | -1.612 |
| Married/cohabiting | -0.044 | 0.014 | -3.21 | 0.001 | -0.071 | -0.017 |
| Children in household | -0.021 | 0.027 | -0.80 | 0.427 | -0.074 | 0.031 |
| Limiting long-standing illness | 0.306 | 0.035 | 8.80 | <0.001 | 0.238 | 0.375 |
| Discrimination exposure | -0.001 | 0.017 | -0.08 | 0.935 | -0.034 | 0.031 |
| Above median income | -0.006 | 0.018 | -0.34 | 0.731 | -0.042 | 0.029 |
| Employed or student | 0.112 | 0.026 | 4.37 | <0.001 | 0.062 | 0.162 |
| Higher education | 0.011 | 0.009 | 1.28 | 0.202 | -0.006 | 0.029 |
| Home ownership | -0.092 | 0.024 | -3.93 | <0.001 | -0.139 | -0.046 |
| Rural residence | 0.032 | 0.026 | 1.24 | 0.215 | -0.018 | 0.082 |
| Neighbourhood cohesion | -0.051 | 0.027 | -1.88 | 0.060 | -0.105 | 0.002 |
|  |  |  |  |  |  |  |
| **Unexplained** |  |  |  |  |  |  |
| Female | 0.074 | 0.141 | 0.52 | 0.601 | -0.203 | 0.351 |
| Age | -3.892 | 1.901 | -2.05 | 0.041 | -7.618 | -0.165 |
| Age squared | 1.862 | 0.986 | 1.89 | 0.059 | -0.070 | 3.795 |
| Married/cohabiting | 0.167 | 0.212 | 0.79 | 0.431 | -0.249 | 0.582 |
| Children in household | 0.154 | 0.137 | 1.13 | 0.259 | -0.114 | 0.423 |
| Limiting long-standing illness | -0.167 | 0.067 | -2.48 | 0.013 | -0.298 | -0.035 |
| Discrimination exposure | 0.021 | 0.069 | 0.30 | 0.765 | -0.114 | 0.155 |
| Above median income | -0.001 | 0.126 | -0.01 | 0.995 | -0.249 | 0.247 |
| Employed or student | -0.090 | 0.278 | -0.32 | 0.745 | -0.635 | 0.455 |
| Higher education | 0.467 | 0.137 | 3.42 | <0.001 | 0.199 | 0.735 |
| Home ownership | -0.460 | 0.215 | -2.14 | 0.032 | -0.881 | -0.039 |
| Rural residence | -0.004 | 0.044 | -0.08 | 0.933 | -0.089 | 0.082 |
| Neighbourhood cohesion | 1.848 | 0.749 | 2.47 | 0.014 | 0.380 | 3.317 |
| Constant | 0.276 | 1.061 | 0.26 | 0.795 | -1.804 | 2.357 |

*Married/cohabiting: Legally married or cohabiting with partner*

*Children in household: One or more children aged under 16 in household*

*Limiting long-standing illness: Long-standing health condition that limits daily activities*

*Discrimination exposure: Physically attacked or avoided places due to fear of attack based on ethnicity, religion, or other characteristic*

*Above median income: Equivalised household income above survey-weighted population median*

*Employed or student: Currently in employment or full-time education*

*Higher education: Degree-level qualification or other higher education*

*Home ownership: Owns home outright or with mortgage*

*Rural residence: Rural area (ONS classification)*

*Neighbourhood cohesion: Scale of neighbourhood belonging and trust, 1-5 (carry-forward imputed from rotating module waves)*

*Source: UK Household Longitudinal Study (UKHLS), waves 2-15 (2010-2024). Survey-weighted Oaxaca-Blinder decomposition with pooled coefficients. Linearised standard errors account for complex survey design.*

**Table A10.** Oaxaca-Blinder decomposition of SF-12 Mental Component: Asian women vs rest

| **Blinder-Oaxaca decomposition** | | | | | | |
| --- | --- | --- | --- | --- | --- | --- |
| Group 1: Rest of the UK population | | | | Number of observations: 278,182 | | |
| Group 2: Asian women | | | | Model: Linear (pooled) | | |
| **SF-12 Mental Component** | **Coeff.** | **Linearised SE** | **t** | **P>\|t\|** | **[95% Conf.** | **Interval]** |
| **Overall** |  |  |  |  |  |  |
| Group 1 mean | 47.717 | 0.120 | 397.60 | <0.001 | 47.482 | 47.952 |
| Group 2 mean | 46.272 | 0.475 | 97.47 | <0.001 | 45.341 | 47.202 |
| Difference | 1.446 | 0.482 | 3.00 | 0.003 | 0.500 | 2.391 |
| Explained | 0.688 | 0.199 | 3.46 | <0.001 | 0.299 | 1.077 |
| Unexplained | 0.758 | 0.435 | 1.74 | 0.082 | -0.095 | 1.611 |
|  |  |  |  |  |  |  |
| **Explained** |  |  |  |  |  |  |
| Age | -1.619 | 0.308 | -5.25 | <0.001 | -2.223 | -1.014 |
| Age squared | 2.988 | 0.368 | 8.12 | <0.001 | 2.267 | 3.709 |
| Married/cohabiting | -0.063 | 0.045 | -1.40 | 0.163 | -0.152 | 0.026 |
| Children in household | 0.058 | 0.058 | 0.99 | 0.321 | -0.057 | 0.173 |
| Limiting long-standing illness | -0.395 | 0.084 | -4.71 | <0.001 | -0.560 | -0.231 |
| Discrimination exposure | 0.107 | 0.058 | 1.85 | 0.064 | -0.006 | 0.220 |
| Above median income | -0.088 | 0.031 | -2.83 | 0.005 | -0.149 | -0.027 |
| Employed or student | -0.027 | 0.033 | -0.83 | 0.409 | -0.091 | 0.037 |
| Higher education | -0.006 | 0.012 | -0.48 | 0.633 | -0.029 | 0.018 |
| Home ownership | -0.064 | 0.033 | -1.92 | 0.055 | -0.129 | 0.001 |
| Rural residence | -0.036 | 0.043 | -0.84 | 0.400 | -0.119 | 0.048 |
| Neighbourhood cohesion | -0.168 | 0.071 | -2.37 | 0.018 | -0.307 | -0.029 |
|  |  |  |  |  |  |  |
| **Unexplained** |  |  |  |  |  |  |
| Age | 10.585 | 5.010 | 2.11 | 0.035 | 0.764 | 20.406 |
| Age squared | -5.048 | 2.514 | -2.01 | 0.045 | -9.975 | -0.120 |
| Married/cohabiting | -0.485 | 0.545 | -0.89 | 0.373 | -1.553 | 0.583 |
| Children in household | -0.607 | 0.417 | -1.46 | 0.145 | -1.425 | 0.210 |
| Limiting long-standing illness | 0.294 | 0.134 | 2.19 | 0.028 | 0.031 | 0.556 |
| Discrimination exposure | 0.062 | 0.197 | 0.31 | 0.754 | -0.325 | 0.449 |
| Above median income | -0.315 | 0.313 | -1.01 | 0.314 | -0.929 | 0.299 |
| Employed or student | 1.292 | 0.557 | 2.32 | 0.020 | 0.201 | 2.384 |
| Higher education | -1.030 | 0.413 | -2.49 | 0.013 | -1.840 | -0.220 |
| Home ownership | 1.385 | 0.712 | 1.94 | 0.052 | -0.012 | 2.781 |
| Rural residence | 0.006 | 0.079 | 0.07 | 0.943 | -0.150 | 0.161 |
| Neighbourhood cohesion | -2.725 | 2.002 | -1.36 | 0.173 | -6.650 | 1.199 |
| Constant | -2.655 | 3.301 | -0.80 | 0.421 | -9.126 | 3.815 |

*Married/cohabiting: Legally married or cohabiting with partner*

*Children in household: One or more children aged under 16 in household*

*Limiting long-standing illness: Long-standing health condition that limits daily activities*

*Discrimination exposure: Physically attacked or avoided places due to fear of attack based on ethnicity, religion, or other characteristic*

*Above median income: Equivalised household income above survey-weighted population median*

*Employed or student: Currently in employment or full-time education*

*Higher education: Degree-level qualification or other higher education*

*Home ownership: Owns home outright or with mortgage*

*Rural residence: Rural area (ONS classification)*

*Neighbourhood cohesion: Scale of neighbourhood belonging and trust, 1-5 (carry-forward imputed from rotating module waves)*

*Source: UK Household Longitudinal Study (UKHLS), waves 2-15 (2010-2024). Survey-weighted Oaxaca-Blinder decomposition with pooled coefficients. Linearised standard errors account for complex survey design.*

**Table A11.** Oaxaca-Blinder decomposition of GHQ-12 Likert: Asian women vs rest

| **Blinder-Oaxaca decomposition** | | | | | | |
| --- | --- | --- | --- | --- | --- | --- |
| Group 1: Rest of the UK population | | | | Number of observations: 278,832 | | |
| Group 2: Asian women | | | | Model: Linear (pooled) | | |
| **GHQ-12 Likert** | **Coeff.** | **Linearised SE** | **t** | **P>\|t\|** | **[95% Conf.** | **Interval]** |
| **Overall** |  |  |  |  |  |  |
| Group 1 mean | 11.593 | 0.061 | 189.49 | <0.001 | 11.473 | 11.713 |
| Group 2 mean | 12.304 | 0.262 | 47.02 | <0.001 | 11.791 | 12.817 |
| Difference | -0.711 | 0.265 | -2.69 | 0.007 | -1.230 | -0.192 |
| Explained | -0.025 | 0.102 | -0.24 | 0.809 | -0.225 | 0.176 |
| Unexplained | -0.686 | 0.236 | -2.90 | 0.004 | -1.150 | -0.223 |
|  |  |  |  |  |  |  |
| **Explained** |  |  |  |  |  |  |
| Age | 1.455 | 0.190 | 7.67 | <0.001 | 1.083 | 1.827 |
| Age squared | -1.874 | 0.207 | -9.06 | <0.001 | -2.279 | -1.468 |
| Married/cohabiting | 0.027 | 0.018 | 1.46 | 0.144 | -0.009 | 0.063 |
| Children in household | -0.035 | 0.032 | -1.12 | 0.265 | -0.098 | 0.027 |
| Limiting long-standing illness | 0.259 | 0.055 | 4.74 | <0.001 | 0.152 | 0.366 |
| Discrimination exposure | -0.047 | 0.028 | -1.72 | 0.086 | -0.101 | 0.007 |
| Above median income | 0.007 | 0.016 | 0.41 | 0.682 | -0.025 | 0.038 |
| Employed or student | 0.015 | 0.019 | 0.81 | 0.419 | -0.021 | 0.051 |
| Higher education | 0.002 | 0.004 | 0.55 | 0.579 | -0.006 | 0.011 |
| Home ownership | 0.032 | 0.016 | 1.95 | 0.051 | -0.000 | 0.064 |
| Rural residence | 0.046 | 0.022 | 2.07 | 0.039 | 0.002 | 0.090 |
| Neighbourhood cohesion | 0.089 | 0.038 | 2.36 | 0.018 | 0.015 | 0.163 |
|  |  |  |  |  |  |  |
| **Unexplained** |  |  |  |  |  |  |
| Age | -4.092 | 2.724 | -1.50 | 0.133 | -9.433 | 1.249 |
| Age squared | 2.001 | 1.345 | 1.49 | 0.137 | -0.635 | 4.637 |
| Married/cohabiting | 0.351 | 0.316 | 1.11 | 0.266 | -0.268 | 0.971 |
| Children in household | 0.280 | 0.216 | 1.29 | 0.196 | -0.144 | 0.703 |
| Limiting long-standing illness | -0.293 | 0.090 | -3.26 | 0.001 | -0.468 | -0.117 |
| Discrimination exposure | -0.114 | 0.111 | -1.03 | 0.301 | -0.332 | 0.103 |
| Above median income | 0.095 | 0.172 | 0.55 | 0.581 | -0.242 | 0.431 |
| Employed or student | -0.450 | 0.310 | -1.45 | 0.147 | -1.058 | 0.159 |
| Higher education | 0.705 | 0.212 | 3.32 | <0.001 | 0.289 | 1.121 |
| Home ownership | -0.553 | 0.395 | -1.40 | 0.161 | -1.326 | 0.221 |
| Rural residence | -0.009 | 0.048 | -0.19 | 0.848 | -0.104 | 0.085 |
| Neighbourhood cohesion | 2.392 | 1.133 | 2.11 | 0.035 | 0.171 | 4.614 |
| Constant | -0.999 | 1.885 | -0.53 | 0.596 | -4.695 | 2.696 |

*Married/cohabiting: Legally married or cohabiting with partner*

*Children in household: One or more children aged under 16 in household*

*Limiting long-standing illness: Long-standing health condition that limits daily activities*

*Discrimination exposure: Physically attacked or avoided places due to fear of attack based on ethnicity, religion, or other characteristic*

*Above median income: Equivalised household income above survey-weighted population median*

*Employed or student: Currently in employment or full-time education*

*Higher education: Degree-level qualification or other higher education*

*Home ownership: Owns home outright or with mortgage*

*Rural residence: Rural area (ONS classification)*

*Neighbourhood cohesion: Scale of neighbourhood belonging and trust, 1-5 (carry-forward imputed from rotating module waves)*

*Source: UK Household Longitudinal Study (UKHLS), waves 2-15 (2010-2024). Survey-weighted Oaxaca-Blinder decomposition with pooled coefficients. Linearised standard errors account for complex survey design.*

**Table A12.** Oaxaca-Blinder decomposition of SF-12 Mental Component: Black women vs rest

| **Blinder-Oaxaca decomposition** | | | | | | |
| --- | --- | --- | --- | --- | --- | --- |
| Group 1: Rest of the UK population | | | | Number of observations: 263,526 | | |
| Group 2: Black women | | | | Model: Linear (pooled) | | |
| **SF-12 Mental Component** | **Coeff.** | **Linearised SE** | **t** | **P>\|t\|** | **[95% Conf.** | **Interval]** |
| **Overall** |  |  |  |  |  |  |
| Group 1 mean | 47.653 | 0.120 | 398.13 | <0.001 | 47.419 | 47.888 |
| Group 2 mean | 47.098 | 0.684 | 68.89 | <0.001 | 45.758 | 48.438 |
| Difference | 0.555 | 0.694 | 0.80 | 0.423 | -0.805 | 1.915 |
| Explained | 2.038 | 0.330 | 6.17 | <0.001 | 1.391 | 2.686 |
| Unexplained | -1.483 | 0.590 | -2.52 | 0.012 | -2.639 | -0.327 |
|  |  |  |  |  |  |  |
| **Explained** |  |  |  |  |  |  |
| Age | -0.959 | 0.232 | -4.14 | <0.001 | -1.414 | -0.505 |
| Age squared | 1.894 | 0.337 | 5.62 | <0.001 | 1.233 | 2.555 |
| Married/cohabiting | 0.492 | 0.084 | 5.83 | <0.001 | 0.326 | 0.657 |
| Children in household | 0.067 | 0.052 | 1.30 | 0.192 | -0.034 | 0.168 |
| Limiting long-standing illness | -0.232 | 0.130 | -1.79 | 0.074 | -0.487 | 0.022 |
| Discrimination exposure | 0.085 | 0.085 | 1.00 | 0.318 | -0.082 | 0.251 |
| Above median income | -0.090 | 0.037 | -2.46 | 0.014 | -0.161 | -0.018 |
| Employed or student | -0.160 | 0.048 | -3.32 | <0.001 | -0.254 | -0.065 |
| Higher education | -0.028 | 0.020 | -1.42 | 0.156 | -0.067 | 0.011 |
| Home ownership | 0.542 | 0.112 | 4.83 | <0.001 | 0.322 | 0.763 |
| Rural residence | -0.023 | 0.048 | -0.47 | 0.638 | -0.118 | 0.072 |
| Neighbourhood cohesion | 0.450 | 0.105 | 4.28 | <0.001 | 0.244 | 0.656 |
|  |  |  |  |  |  |  |
| **Unexplained** |  |  |  |  |  |  |
| Age | 7.944 | 6.533 | 1.22 | 0.224 | -4.864 | 20.751 |
| Age squared | -6.396 | 3.599 | -1.78 | 0.076 | -13.452 | 0.659 |
| Married/cohabiting | -0.104 | 0.352 | -0.30 | 0.767 | -0.794 | 0.586 |
| Children in household | -0.549 | 0.611 | -0.90 | 0.369 | -1.746 | 0.648 |
| Limiting long-standing illness | -0.031 | 0.255 | -0.12 | 0.903 | -0.532 | 0.469 |
| Discrimination exposure | -0.072 | 0.253 | -0.29 | 0.776 | -0.568 | 0.424 |
| Above median income | 0.872 | 0.453 | 1.93 | 0.054 | -0.016 | 1.760 |
| Employed or student | -2.419 | 1.182 | -2.05 | 0.041 | -4.735 | -0.102 |
| Higher education | -1.515 | 0.603 | -2.51 | 0.012 | -2.698 | -0.332 |
| Home ownership | -0.134 | 0.373 | -0.36 | 0.719 | -0.865 | 0.596 |
| Rural residence | 0.051 | 0.051 | 0.98 | 0.325 | -0.050 | 0.152 |
| Neighbourhood cohesion | 1.574 | 2.764 | 0.57 | 0.569 | -3.845 | 6.992 |
| Constant | -0.702 | 3.384 | -0.21 | 0.836 | -7.336 | 5.931 |

*Married/cohabiting: Legally married or cohabiting with partner*

*Children in household: One or more children aged under 16 in household*

*Limiting long-standing illness: Long-standing health condition that limits daily activities*

*Discrimination exposure: Physically attacked or avoided places due to fear of attack based on ethnicity, religion, or other characteristic*

*Above median income: Equivalised household income above survey-weighted population median*

*Employed or student: Currently in employment or full-time education*

*Higher education: Degree-level qualification or other higher education*

*Home ownership: Owns home outright or with mortgage*

*Rural residence: Rural area (ONS classification)*

*Neighbourhood cohesion: Scale of neighbourhood belonging and trust, 1-5 (carry-forward imputed from rotating module waves)*

*Source: UK Household Longitudinal Study (UKHLS), waves 2-15 (2010-2024). Survey-weighted Oaxaca-Blinder decomposition with pooled coefficients. Linearised standard errors account for complex survey design.*

**Table A13.** Oaxaca-Blinder decomposition of GHQ-12 Likert: Black women vs rest

| **Blinder-Oaxaca decomposition** | | | | | | |
| --- | --- | --- | --- | --- | --- | --- |
| Group 1: Rest of the UK population | | | | Number of observations: 264,239 | | |
| Group 2: Black women | | | | Model: Linear (pooled) | | |
| **GHQ-12 Likert** | **Coeff.** | **Linearised SE** | **t** | **P>\|t\|** | **[95% Conf.** | **Interval]** |
| **Overall** |  |  |  |  |  |  |
| Group 1 mean | 11.633 | 0.061 | 192.13 | <0.001 | 11.514 | 11.751 |
| Group 2 mean | 11.670 | 0.416 | 28.07 | <0.001 | 10.855 | 12.485 |
| Difference | -0.037 | 0.418 | -0.09 | 0.929 | -0.856 | 0.782 |
| Explained | -0.824 | 0.171 | -4.83 | <0.001 | -1.159 | -0.490 |
| Unexplained | 0.787 | 0.371 | 2.12 | 0.034 | 0.060 | 1.514 |
|  |  |  |  |  |  |  |
| **Explained** |  |  |  |  |  |  |
| Age | 0.855 | 0.171 | 5.00 | <0.001 | 0.520 | 1.190 |
| Age squared | -1.184 | 0.201 | -5.88 | <0.001 | -1.579 | -0.789 |
| Married/cohabiting | -0.198 | 0.040 | -4.89 | <0.001 | -0.277 | -0.118 |
| Children in household | -0.042 | 0.028 | -1.52 | 0.130 | -0.096 | 0.012 |
| Limiting long-standing illness | 0.147 | 0.084 | 1.74 | 0.082 | -0.019 | 0.312 |
| Discrimination exposure | -0.040 | 0.040 | -1.01 | 0.314 | -0.119 | 0.038 |
| Above median income | -0.002 | 0.019 | -0.08 | 0.938 | -0.040 | 0.037 |
| Employed or student | 0.089 | 0.027 | 3.30 | <0.001 | 0.036 | 0.142 |
| Higher education | 0.009 | 0.008 | 1.09 | 0.276 | -0.007 | 0.025 |
| Home ownership | -0.261 | 0.058 | -4.53 | <0.001 | -0.374 | -0.148 |
| Rural residence | 0.040 | 0.025 | 1.59 | 0.112 | -0.009 | 0.089 |
| Neighbourhood cohesion | -0.237 | 0.055 | -4.32 | <0.001 | -0.344 | -0.129 |
|  |  |  |  |  |  |  |
| **Unexplained** |  |  |  |  |  |  |
| Age | -0.184 | 3.693 | -0.05 | 0.960 | -7.424 | 7.055 |
| Age squared | 0.780 | 1.775 | 0.44 | 0.660 | -2.700 | 4.260 |
| Married/cohabiting | 0.395 | 0.262 | 1.51 | 0.132 | -0.118 | 0.907 |
| Children in household | -0.118 | 0.374 | -0.32 | 0.751 | -0.851 | 0.615 |
| Limiting long-standing illness | 0.135 | 0.156 | 0.86 | 0.387 | -0.171 | 0.441 |
| Discrimination exposure | 0.027 | 0.163 | 0.17 | 0.867 | -0.293 | 0.347 |
| Above median income | -0.425 | 0.285 | -1.49 | 0.136 | -0.984 | 0.134 |
| Employed or student | 0.345 | 0.578 | 0.60 | 0.550 | -0.787 | 1.478 |
| Higher education | 0.572 | 0.407 | 1.41 | 0.160 | -0.226 | 1.370 |
| Home ownership | 0.002 | 0.195 | 0.01 | 0.994 | -0.382 | 0.385 |
| Rural residence | 0.006 | 0.017 | 0.39 | 0.697 | -0.026 | 0.039 |
| Neighbourhood cohesion | 0.402 | 2.097 | 0.19 | 0.848 | -3.708 | 4.512 |
| Constant | -1.150 | 2.334 | -0.49 | 0.622 | -5.725 | 3.426 |

*Married/cohabiting: Legally married or cohabiting with partner*

*Children in household: One or more children aged under 16 in household*

*Limiting long-standing illness: Long-standing health condition that limits daily activities*

*Discrimination exposure: Physically attacked or avoided places due to fear of attack based on ethnicity, religion, or other characteristic*

*Above median income: Equivalised household income above survey-weighted population median*

*Employed or student: Currently in employment or full-time education*

*Higher education: Degree-level qualification or other higher education*

*Home ownership: Owns home outright or with mortgage*

*Rural residence: Rural area (ONS classification)*

*Neighbourhood cohesion: Scale of neighbourhood belonging and trust, 1-5 (carry-forward imputed from rotating module waves)*

*Source: UK Household Longitudinal Study (UKHLS), waves 2-15 (2010-2024). Survey-weighted Oaxaca-Blinder decomposition with pooled coefficients. Linearised standard errors account for complex survey design.*

**Table A14.** Oaxaca-Blinder decomposition of SF-12 Mental Component: Model M9 — robustness check with pidp-level clustering (ethnic minority women vs rest of population)

| **Blinder-Oaxaca decomposition** | | | | | | |
| --- | --- | --- | --- | --- | --- | --- |
| Group 1: Rest of the UK population | | | | Number of observations: 291,711 | | |
| Group 2: Ethnic minority women | | | | Model: Linear (pooled) | | |
| **SF-12 Mental Component** | **Coeff.** | **Linearised SE** | **t** | **P>\|t\|** | **[95% Conf.** | **Interval]** |
| **Overall** |  |  |  |  |  |  |
| Group 1 mean | 47.854 | 0.113 | 422.30 | <0.001 | 47.632 | 48.076 |
| Group 2 mean | 46.023 | 0.357 | 128.85 | <0.001 | 45.323 | 46.723 |
| Difference | 1.831 | 0.375 | 4.89 | <0.001 | 1.096 | 2.565 |
| Explained | 1.547 | 0.173 | 8.93 | <0.001 | 1.207 | 1.886 |
| Unexplained | 0.284 | 0.348 | 0.82 | 0.414 | -0.397 | 0.966 |
|  |  |  |  |  |  |  |
| **Explained** |  |  |  |  |  |  |
| Age | -1.520 | 0.289 | -5.26 | <0.001 | -2.086 | -0.953 |
| Age squared | 2.847 | 0.330 | 8.63 | <0.001 | 2.201 | 3.494 |
| Married/cohabiting | 0.198 | 0.043 | 4.63 | <0.001 | 0.114 | 0.281 |
| Children in household | 0.051 | 0.047 | 1.08 | 0.279 | -0.041 | 0.144 |
| Limiting long-standing illness | -0.250 | 0.071 | -3.50 | <0.001 | -0.390 | -0.110 |
| Discrimination exposure | 0.108 | 0.045 | 2.37 | 0.018 | 0.019 | 0.197 |
| Above median income | -0.088 | 0.030 | -2.92 | 0.004 | -0.147 | -0.029 |
| Employed or student | -0.104 | 0.030 | -3.44 | <0.001 | -0.163 | -0.045 |
| Higher education | -0.018 | 0.012 | -1.51 | 0.130 | -0.042 | 0.005 |
| Home ownership | 0.193 | 0.044 | 4.42 | <0.001 | 0.107 | 0.278 |
| Rural residence | -0.034 | 0.039 | -0.87 | 0.385 | -0.112 | 0.043 |
| Neighbourhood cohesion | 0.164 | 0.063 | 2.58 | 0.010 | 0.039 | 0.288 |
|  |  |  |  |  |  |  |
| **Unexplained** |  |  |  |  |  |  |
| Age | 8.351 | 4.224 | 1.98 | 0.048 | 0.072 | 16.630 |
| Age squared | -5.210 | 2.236 | -2.33 | 0.020 | -9.593 | -0.828 |
| Married/cohabiting | -0.360 | 0.350 | -1.03 | 0.303 | -1.045 | 0.325 |
| Children in household | -0.598 | 0.310 | -1.93 | 0.054 | -1.206 | 0.010 |
| Limiting long-standing illness | 0.280 | 0.142 | 1.98 | 0.048 | 0.002 | 0.557 |
| Discrimination exposure | -0.082 | 0.155 | -0.53 | 0.598 | -0.386 | 0.222 |
| Above median income | 0.512 | 0.281 | 1.82 | 0.068 | -0.039 | 1.064 |
| Employed or student | 0.193 | 0.551 | 0.35 | 0.726 | -0.887 | 1.273 |
| Higher education | -1.193 | 0.320 | -3.73 | <0.001 | -1.820 | -0.565 |
| Home ownership | 1.052 | 0.397 | 2.65 | 0.008 | 0.274 | 1.830 |
| Rural residence | 0.093 | 0.091 | 1.02 | 0.307 | -0.085 | 0.270 |
| Neighbourhood cohesion | -1.899 | 1.627 | -1.17 | 0.243 | -5.088 | 1.289 |
| Constant | -0.855 | 2.551 | -0.33 | 0.738 | -5.855 | 4.146 |

*Married/cohabiting: Legally married or cohabiting with partner*

*Children in household: One or more children aged under 16 in household*

*Limiting long-standing illness: Long-standing health condition that limits daily activities*

*Discrimination exposure: Physically attacked or avoided places due to fear of attack based on ethnicity, religion, or other characteristic*

*Above median income: Equivalised household income above survey-weighted population median*

*Employed or student: Currently in employment or full-time education*

*Higher education: Degree-level qualification or other higher education*

*Home ownership: Owns home outright or with mortgage*

*Rural residence: Rural area (ONS classification)*

*Neighbourhood cohesion: Scale of neighbourhood belonging and trust, 1-5 (carry-forward imputed from rotating module waves)*

*Source: UK Household Longitudinal Study (UKHLS), waves 2-15 (2010-2024). Survey-weighted Oaxaca-Blinder decomposition with pooled coefficients. Linearised standard errors account for complex survey design.*

**Table A15.** Sample attrition flow for the M1 analytical samples (SF-12 and GHQ-12 outcomes)

| **Attrition flow from raw merged dataset to analytical samples** | | | | | |
| --- | --- | --- | --- | --- | --- |
| **Step** | **Description** | **SF-12 Mental Component sample** | | **GHQ-12 Likert sample** | |
|  |  | **N excluded** | **N remaining** | **N excluded** | **N remaining** |
| 0 | Raw merged dataset (all 15 waves) | 0 | 594,342 | 0 | 594,342 |
| 1 | Drop wave 1 | 50,994 | 543,348 | 50,994 | 543,348 |
| 2 | Drop missing weight_lw | 0 | 543,348 | 0 | 543,348 |
| 3 | Drop missing outcome (SF-12 Mental Health) | 68,683 | 474,665 | 60,258 | 483,090 |
| 4 | Drop missing group var (non_white_fem) | 418 | 474,247 | 422 | 482,668 |
| 5 | Apply M1 covariate vector (oaxaca svy listwise) | 182,536 | 291,711 | 190,082 | 292,586 |

*Source: UKHLS waves 1-15 (raw merged dataset, 594,342 person-wave observations). SF-12 final analytical sample N = 291,711; GHQ-12 final analytical sample N = 292,586. Step 5 reflects the listwise compaction performed by the M1 oaxaca command on its full covariate vector (including dscr2 and nei after carry-forward imputation).*

**Table A16.** Oaxaca-Blinder decomposition of GHQ-12 Likert: Model M9 — robustness check with pidp-level clustering (ethnic minority women vs rest of population)

| **Blinder-Oaxaca decomposition** | | | | | | |
| --- | --- | --- | --- | --- | --- | --- |
| Group 1: Rest of the UK population | | | | Number of observations: 292,586 | | |
| Group 2: Ethnic minority women | | | | Model: Linear (pooled) | | |
| **GHQ-12 Likert** | **Coeff.** | **Linearised SE** | **t** | **P>\|t\|** | **[95% Conf.** | **Interval]** |
| **Overall** |  |  |  |  |  |  |
| Group 1 mean | 11.536 | 0.057 | 201.19 | <0.001 | 11.424 | 11.649 |
| Group 2 mean | 12.348 | 0.207 | 59.77 | <0.001 | 11.943 | 12.753 |
| Difference | -0.811 | 0.215 | -3.78 | <0.001 | -1.232 | -0.391 |
| Explained | -0.466 | 0.088 | -5.29 | <0.001 | -0.639 | -0.293 |
| Unexplained | -0.346 | 0.201 | -1.72 | 0.085 | -0.739 | 0.048 |
|  |  |  |  |  |  |  |
| **Explained** |  |  |  |  |  |  |
| Age | 1.367 | 0.172 | 7.95 | <0.001 | 1.030 | 1.703 |
| Age squared | -1.783 | 0.183 | -9.73 | <0.001 | -2.142 | -1.424 |
| Married/cohabiting | -0.077 | 0.019 | -4.05 | <0.001 | -0.114 | -0.039 |
| Children in household | -0.030 | 0.025 | -1.21 | 0.227 | -0.080 | 0.019 |
| Limiting long-standing illness | 0.165 | 0.046 | 3.59 | <0.001 | 0.075 | 0.255 |
| Discrimination exposure | -0.049 | 0.022 | -2.28 | 0.023 | -0.092 | -0.007 |
| Above median income | 0.006 | 0.016 | 0.36 | 0.716 | -0.026 | 0.038 |
| Employed or student | 0.058 | 0.017 | 3.47 | <0.001 | 0.025 | 0.091 |
| Higher education | 0.007 | 0.006 | 1.25 | 0.213 | -0.004 | 0.018 |
| Home ownership | -0.090 | 0.022 | -4.12 | <0.001 | -0.133 | -0.047 |
| Rural residence | 0.047 | 0.021 | 2.21 | 0.027 | 0.005 | 0.088 |
| Neighbourhood cohesion | -0.085 | 0.033 | -2.56 | 0.010 | -0.151 | -0.020 |
|  |  |  |  |  |  |  |
| **Unexplained** |  |  |  |  |  |  |
| Age | -2.476 | 2.482 | -1.00 | 0.318 | -7.342 | 2.389 |
| Age squared | 1.591 | 1.275 | 1.25 | 0.212 | -0.907 | 4.090 |
| Married/cohabiting | 0.287 | 0.214 | 1.34 | 0.181 | -0.134 | 0.707 |
| Children in household | 0.116 | 0.183 | 0.64 | 0.525 | -0.242 | 0.474 |
| Limiting long-standing illness | -0.213 | 0.092 | -2.31 | 0.021 | -0.394 | -0.032 |
| Discrimination exposure | -0.042 | 0.091 | -0.46 | 0.645 | -0.221 | 0.137 |
| Above median income | -0.243 | 0.167 | -1.46 | 0.144 | -0.570 | 0.083 |
| Employed or student | -0.068 | 0.296 | -0.23 | 0.818 | -0.649 | 0.512 |
| Higher education | 0.660 | 0.186 | 3.55 | <0.001 | 0.296 | 1.025 |
| Home ownership | -0.576 | 0.230 | -2.50 | 0.012 | -1.027 | -0.125 |
| Rural residence | -0.085 | 0.057 | -1.49 | 0.137 | -0.196 | 0.027 |
| Neighbourhood cohesion | 2.041 | 1.032 | 1.98 | 0.048 | 0.019 | 4.063 |
| Constant | -1.337 | 1.538 | -0.87 | 0.385 | -4.351 | 1.677 |

*Married/cohabiting: Legally married or cohabiting with partner*

*Children in household: One or more children aged under 16 in household*

*Limiting long-standing illness: Long-standing health condition that limits daily activities*

*Discrimination exposure: Physically attacked or avoided places due to fear of attack based on ethnicity, religion, or other characteristic*

*Above median income: Equivalised household income above survey-weighted population median*

*Employed or student: Currently in employment or full-time education*

*Higher education: Degree-level qualification or other higher education*

*Home ownership: Owns home outright or with mortgage*

*Rural residence: Rural area (ONS classification)*

*Neighbourhood cohesion: Scale of neighbourhood belonging and trust, 1-5 (carry-forward imputed from rotating module waves)*

*Source: UK Household Longitudinal Study (UKHLS), waves 2-15 (2010-2024). Survey-weighted Oaxaca-Blinder decomposition with pooled coefficients. Linearised standard errors account for complex survey design.*

**Table A17.** Composition of the M3 (observed-only) versus M1 (carry-forward) analytical samples

| **M1 carry-forward sample vs M3 observed-only sample** | | | | |
| --- | --- | --- | --- | --- |
| **Variable** | **M1 (mean / %)** | **M3 (mean / %)** | **Difference (M3 − M1)** | **Direction** |
| Age (years) | 48.48 | 46.67 | -1.82 | ↓ |
| Ethnic minority women (%) | 12.7 | 17.9 | +5.2 pp | ↑ |
| Female (%) | 53.5 | 53.0 | -0.5 pp | ↓ |
| Higher education (%) | 40.2 | 39.4 | -0.8 pp | ↓ |
| Above median income (%) | 59.8 | 56.4 | -3.4 pp | ↓ |
| Home ownership (%) | 71.4 | 69.2 | -2.2 pp | ↓ |
| Married/cohabiting (%) | 63.3 | 61.8 | -1.5 pp | ↓ |
| Limiting long-standing illness (%) | 22.0 | 21.3 | -0.8 pp | ↓ |
| Neighbourhood cohesion (1-5) | 3.54 | 3.54 | -0.00 | ↓ |
| Discrimination exposure (%) | 20.5 | 19.7 | -0.7 pp | ↓ |

*Survey-weighted means (continuous) or shares (binary). M1 sample N = 291,711 person-waves; M3 sample N = 175,779 person-waves. The M3 sample is observed-only — it drops person-wave observations whose discrimination or neighbourhood-cohesion values would otherwise be filled by carry-forward — and is consequently slightly younger and has a higher share of ethnic minority women than M1. "pp" denotes percentage-point difference for binary variables.*

**Table A18.** Within-person intraclass correlations (ICCs) from random-intercept null models

| **Intraclass correlations from random-intercept null models** | | | | | |
| --- | --- | --- | --- | --- | --- |
| **Variable** | **ICC** | **95% CI lower** | **95% CI upper** | **Person-wave N** | **Unique pidp** |
| SF-12 Mental Component (continuous) | 0.518 | 0.515 | 0.521 | 522,065 | 90,887 |
| GHQ-12 Likert (continuous) | 0.483 | 0.480 | 0.487 | 522,790 | 89,155 |
| Higher education (binary) | 0.926 | 0.925 | 0.926 | 584,897 | 96,208 |
| Home ownership (binary) | 0.872 | 0.871 | 0.873 | 590,873 | 98,899 |
| Employed or student (binary) | 0.727 | 0.725 | 0.729 | 593,712 | 99,077 |
| Above median income (binary) | 0.436 | 0.432 | 0.439 | 594,342 | 99,147 |
| Married/cohabiting (binary) | 0.845 | 0.843 | 0.846 | 593,105 | 99,025 |
| Limiting long-standing illness (binary) | 0.514 | 0.511 | 0.517 | 592,964 | 98,960 |
| Neighbourhood cohesion (continuous, 1-5) | 0.723 | 0.720 | 0.725 | 514,040 | 69,172 |
| Discrimination exposure (binary) | 0.745 | 0.742 | 0.748 | 226,419 | 41,671 |

*Random-intercept null model fit per variable: mixed [outcome] || pidp: . For continuous outcomes the ICC is computed directly from the variance components (σ²_pidp / (σ²_pidp + σ²_resid)). For binary variables the linear-probability-model ICC is reported, with its standard error obtained via the delta method on the variance-ratio form. ICCs > 0.5 indicate that more than half of the variance is between-person, motivating cluster-robust inference (M9, Table A14 and A16) and informing the carry-forward design for time-stable covariates.*

**Table A19.** Source of discrimination (dscr2) and neighbourhood cohesion (nei) values in the M1 analytical sample

| **Source of imputed values: directly observed, carry-forward, carry-back** | | | | |
| --- | --- | --- | --- | --- |
| **Variable** | **Directly observed** | **Carry-forward (mean wave-distance)** | **Carry-back (mean wave-distance)** | **Total non-missing in M1** |
| dscr2 (discrimination) | 43,985 (27.6%) | 87,959 (55.2%; mean dist. 2.2 waves) | 25,363 (15.9%; mean dist. 1.2 waves) | 159,481 |
| nei (neighbourhood cohesion) | 69,341 (26.5%) | 139,108 (53.1%; mean dist. 1.9 waves) | 41,423 (15.8%; mean dist. 1.6 waves) | 261,829 |

*M1 sample (N = 291,711 person-waves). Discrimination (dscr2) is fielded in UKHLS rotating modules and neighbourhood cohesion (nei) in cross-module rotation; values are missing in waves where the module was not asked. Carry-forward fills a missing wave-t value with the most recent observed value within person; carry-back fills with the next observed value when no prior value exists. Mean wave-distance is the average gap (in waves) between the imputed wave and the donor wave. Percentages within each row do not sum exactly to 100% (residual: dscr2 (discrimination) 1.4%, nei (neighbourhood cohesion) 4.6%); the residual reflects person-wave observations resolved by panel-edge rules in the imputation routine that fall outside the carry-forward / carry-back classification.*

**Table A20.** Distribution of waves contributed per individual to the M1 SF-12 analytical sample

| **Waves contributed per individual (M1 SF-12 sample)** | | |
| --- | --- | --- |
| **Waves contributed** | **Number of individuals** | **% of unique individuals** |
| 1 | 8,939 | 17.76 |
| 2 | 5,743 | 11.41 |
| 3 | 4,439 | 8.82 |
| 4 | 4,135 | 8.21 |
| 5 | 3,804 | 7.56 |
| 6 | 6,105 | 12.13 |
| 7 | 2,151 | 4.27 |
| 8 | 1,902 | 3.78 |
| 9 | 1,848 | 3.67 |
| 10 | 1,956 | 3.89 |
| 11 | 1,747 | 3.47 |
| 12 | 1,775 | 3.53 |
| 13 | 2,291 | 4.55 |
| 14 | 3,501 | 6.96 |
| **Total** | **50,336** | **100.00** |

*M1 SF-12 sample. N = 291,711 person-wave observations contributed by 50,336 unique individuals. Mean 5.80 waves/person (median 5, range 1-14). UKHLS waves run from 2 (2010-11) through 15 (2023-24) for this sample; wave 1 is excluded because it lacks longitudinal weights.*

**Table A21.** Oaxaca-Blinder decomposition of SF-12 Mental Component: ethnic minority women vs white women (women-only sample)

| **Blinder-Oaxaca decomposition** | | | | | | |
| --- | --- | --- | --- | --- | --- | --- |
| Group 1: White women | | | | Number of observations: 151,770 | | |
| Group 2: Ethnic minority women | | | | Model: Linear (pooled) | | |
| **SF-12 Mental Component** | **Coeff.** | **Linearised SE** | **t** | **P>\|t\|** | **[95% Conf.** | **Interval]** |
| **Overall** |  |  |  |  |  |  |
| Group 1 mean | 46.622 | 0.163 | 285.40 | <0.001 | 46.302 | 46.942 |
| Group 2 mean | 46.023 | 0.364 | 126.30 | <0.001 | 45.309 | 46.738 |
| Difference | 0.599 | 0.400 | 1.50 | 0.134 | -0.184 | 1.382 |
| Explained | 1.644 | 0.206 | 7.99 | <0.001 | 1.241 | 2.048 |
| Unexplained | -1.046 | 0.381 | -2.75 | 0.006 | -1.792 | -0.299 |
|  |  |  |  |  |  |  |
| **Explained** |  |  |  |  |  |  |
| Age | -0.996 | 0.411 | -2.43 | 0.015 | -1.801 | -0.191 |
| Age squared | 2.952 | 0.448 | 6.59 | <0.001 | 2.074 | 3.831 |
| Married/cohabiting | 0.141 | 0.043 | 3.26 | 0.001 | 0.056 | 0.226 |
| Children in household | -0.010 | 0.068 | -0.14 | 0.887 | -0.142 | 0.123 |
| Limiting long-standing illness | -0.449 | 0.075 | -6.00 | <0.001 | -0.596 | -0.303 |
| Discrimination exposure | 0.008 | 0.045 | 0.18 | 0.855 | -0.080 | 0.097 |
| Above median income | -0.138 | 0.042 | -3.30 | <0.001 | -0.220 | -0.056 |
| Employed or student | -0.123 | 0.048 | -2.55 | 0.011 | -0.217 | -0.028 |
| Higher education | -0.060 | 0.026 | -2.28 | 0.022 | -0.112 | -0.009 |
| Home ownership | 0.168 | 0.049 | 3.42 | <0.001 | 0.072 | 0.264 |
| Rural residence | -0.100 | 0.066 | -1.52 | 0.128 | -0.230 | 0.029 |
| Neighbourhood cohesion | 0.251 | 0.067 | 3.72 | <0.001 | 0.119 | 0.383 |
|  |  |  |  |  |  |  |
| **Unexplained** |  |  |  |  |  |  |
| Age | 14.592 | 4.616 | 3.16 | 0.002 | 5.543 | 23.642 |
| Age squared | -7.823 | 2.466 | -3.17 | 0.002 | -12.659 | -2.988 |
| Married/cohabiting | -0.367 | 0.377 | -0.97 | 0.331 | -1.107 | 0.373 |
| Children in household | -0.488 | 0.309 | -1.58 | 0.115 | -1.094 | 0.119 |
| Limiting long-standing illness | 0.358 | 0.156 | 2.30 | 0.022 | 0.053 | 0.663 |
| Discrimination exposure | -0.005 | 0.170 | -0.03 | 0.978 | -0.338 | 0.328 |
| Above median income | 0.507 | 0.315 | 1.61 | 0.107 | -0.110 | 1.123 |
| Employed or student | -0.410 | 0.566 | -0.72 | 0.469 | -1.520 | 0.701 |
| Higher education | -1.006 | 0.320 | -3.14 | 0.002 | -1.633 | -0.378 |
| Home ownership | 1.206 | 0.442 | 2.73 | 0.006 | 0.339 | 2.074 |
| Rural residence | 0.096 | 0.106 | 0.91 | 0.365 | -0.112 | 0.304 |
| Neighbourhood cohesion | -2.663 | 1.738 | -1.53 | 0.125 | -6.069 | 0.744 |
| Constant | -5.043 | 2.730 | -1.85 | 0.065 | -10.396 | 0.310 |

*Married/cohabiting: Legally married or cohabiting with partner*

*Children in household: One or more children aged under 16 in household*

*Limiting long-standing illness: Long-standing health condition that limits daily activities*

*Discrimination exposure: Physically attacked or avoided places due to fear of attack based on ethnicity, religion, or other characteristic*

*Above median income: Equivalised household income above survey-weighted population median*

*Employed or student: Currently in employment or full-time education*

*Higher education: Degree-level qualification or other higher education*

*Home ownership: Owns home outright or with mortgage*

*Rural residence: Rural area (ONS classification)*

*Neighbourhood cohesion: Scale of neighbourhood belonging and trust, 1-5 (carry-forward imputed from rotating module waves)*

*Source: UK Household Longitudinal Study (UKHLS), waves 2-15 (2010-2024). Survey-weighted Oaxaca-Blinder decomposition with pooled coefficients. Linearised standard errors account for complex survey design.*

**Table A22.** Oaxaca-Blinder decomposition of GHQ-12 Likert: ethnic minority women vs white women (women-only sample)

| **Blinder-Oaxaca decomposition** | | | | | | |
| --- | --- | --- | --- | --- | --- | --- |
| Group 1: White women | | | | Number of observations: 152,656 | | |
| Group 2: Ethnic minority women | | | | Model: Linear (pooled) | | |
| **GHQ-12 Likert** | **Coeff.** | **Linearised SE** | **t** | **P>\|t\|** | **[95% Conf.** | **Interval]** |
| **Overall** |  |  |  |  |  |  |
| Group 1 mean | 12.258 | 0.083 | 146.98 | <0.001 | 12.095 | 12.422 |
| Group 2 mean | 12.348 | 0.213 | 58.06 | <0.001 | 11.931 | 12.765 |
| Difference | -0.090 | 0.228 | -0.39 | 0.694 | -0.537 | 0.357 |
| Explained | -0.377 | 0.103 | -3.65 | <0.001 | -0.580 | -0.175 |
| Unexplained | 0.288 | 0.222 | 1.30 | 0.195 | -0.147 | 0.723 |
|  |  |  |  |  |  |  |
| **Explained** |  |  |  |  |  |  |
| Age | 1.288 | 0.233 | 5.54 | <0.001 | 0.832 | 1.744 |
| Age squared | -1.930 | 0.243 | -7.96 | <0.001 | -2.406 | -1.455 |
| Married/cohabiting | -0.055 | 0.019 | -2.87 | 0.004 | -0.092 | -0.017 |
| Children in household | -0.014 | 0.037 | -0.37 | 0.709 | -0.087 | 0.059 |
| Limiting long-standing illness | 0.310 | 0.050 | 6.20 | <0.001 | 0.212 | 0.408 |
| Discrimination exposure | -0.002 | 0.023 | -0.07 | 0.941 | -0.047 | 0.044 |
| Above median income | 0.036 | 0.024 | 1.55 | 0.122 | -0.010 | 0.083 |
| Employed or student | 0.085 | 0.028 | 3.06 | 0.002 | 0.030 | 0.139 |
| Higher education | 0.027 | 0.013 | 2.13 | 0.033 | 0.002 | 0.052 |
| Home ownership | -0.074 | 0.026 | -2.90 | 0.004 | -0.124 | -0.024 |
| Rural residence | 0.090 | 0.037 | 2.40 | 0.016 | 0.016 | 0.163 |
| Neighbourhood cohesion | -0.139 | 0.038 | -3.66 | <0.001 | -0.213 | -0.064 |
|  |  |  |  |  |  |  |
| **Unexplained** |  |  |  |  |  |  |
| Age | -4.947 | 2.636 | -1.88 | 0.061 | -10.115 | 0.220 |
| Age squared | 2.661 | 1.369 | 1.94 | 0.052 | -0.024 | 5.345 |
| Married/cohabiting | 0.303 | 0.225 | 1.34 | 0.179 | -0.139 | 0.745 |
| Children in household | 0.086 | 0.182 | 0.47 | 0.636 | -0.271 | 0.444 |
| Limiting long-standing illness | -0.230 | 0.099 | -2.32 | 0.020 | -0.425 | -0.036 |
| Discrimination exposure | -0.054 | 0.098 | -0.55 | 0.582 | -0.247 | 0.139 |
| Above median income | -0.208 | 0.181 | -1.15 | 0.252 | -0.564 | 0.148 |
| Employed or student | 0.178 | 0.307 | 0.58 | 0.561 | -0.424 | 0.781 |
| Higher education | 0.579 | 0.183 | 3.17 | 0.002 | 0.221 | 0.938 |
| Home ownership | -0.640 | 0.255 | -2.50 | 0.012 | -1.140 | -0.139 |
| Rural residence | -0.093 | 0.066 | -1.42 | 0.157 | -0.221 | 0.036 |
| Neighbourhood cohesion | 2.228 | 1.070 | 2.08 | 0.037 | 0.131 | 4.326 |
| Constant | 0.424 | 1.612 | 0.26 | 0.792 | -2.737 | 3.585 |

*Married/cohabiting: Legally married or cohabiting with partner*

*Children in household: One or more children aged under 16 in household*

*Limiting long-standing illness: Long-standing health condition that limits daily activities*

*Discrimination exposure: Physically attacked or avoided places due to fear of attack based on ethnicity, religion, or other characteristic*

*Above median income: Equivalised household income above survey-weighted population median*

*Employed or student: Currently in employment or full-time education*

*Higher education: Degree-level qualification or other higher education*

*Home ownership: Owns home outright or with mortgage*

*Rural residence: Rural area (ONS classification)*

*Neighbourhood cohesion: Scale of neighbourhood belonging and trust, 1-5 (carry-forward imputed from rotating module waves)*

*Source: UK Household Longitudinal Study (UKHLS), waves 2-15 (2010-2024). Survey-weighted Oaxaca-Blinder decomposition with pooled coefficients. Linearised standard errors account for complex survey design.*

**Table A23.** Oaxaca-Blinder decomposition of SF-12 Mental Component: ethnic minority women vs rest, with log equivalised household income (replacing the binary above-median indicator)

| **Blinder-Oaxaca decomposition** | | | | | | |
| --- | --- | --- | --- | --- | --- | --- |
| Group 1: Rest of the UK population | | | | Number of observations: 277,018 | | |
| Group 2: Ethnic minority women | | | | Model: Linear (pooled) | | |
| **SF-12 Mental Component** | **Coeff.** | **Linearised SE** | **t** | **P>\|t\|** | **[95% Conf.** | **Interval]** |
| **Overall** |  |  |  |  |  |  |
| Group 1 mean | 48.203 | 0.123 | 393.13 | <0.001 | 47.962 | 48.443 |
| Group 2 mean | 46.548 | 0.337 | 138.18 | <0.001 | 45.887 | 47.208 |
| Difference | 1.655 | 0.354 | 4.67 | <0.001 | 0.961 | 2.349 |
| Explained | 1.418 | 0.154 | 9.18 | <0.001 | 1.115 | 1.721 |
| Unexplained | 0.237 | 0.336 | 0.71 | 0.480 | -0.421 | 0.895 |
|  |  |  |  |  |  |  |
| **Explained** |  |  |  |  |  |  |
| Age | -1.566 | 0.278 | -5.63 | <0.001 | -2.110 | -1.021 |
| Age squared | 2.710 | 0.324 | 8.37 | <0.001 | 2.075 | 3.344 |
| Married/cohabiting | 0.168 | 0.039 | 4.37 | <0.001 | 0.093 | 0.244 |
| Children in household | -0.000 | 0.051 | -0.00 | 0.998 | -0.100 | 0.100 |
| Limiting long-standing illness | -0.216 | 0.063 | -3.46 | <0.001 | -0.339 | -0.094 |
| Discrimination exposure | 0.098 | 0.038 | 2.54 | 0.011 | 0.022 | 0.173 |
| Log equivalised HH income | 0.043 | 0.016 | 2.64 | 0.008 | 0.011 | 0.075 |
| Employed or student | -0.058 | 0.026 | -2.23 | 0.026 | -0.109 | -0.007 |
| Higher education | -0.014 | 0.011 | -1.28 | 0.202 | -0.036 | 0.008 |
| Home ownership | 0.148 | 0.043 | 3.45 | <0.001 | 0.064 | 0.232 |
| Rural residence | -0.008 | 0.045 | -0.18 | 0.856 | -0.096 | 0.080 |
| Neighbourhood cohesion | 0.114 | 0.056 | 2.05 | 0.041 | 0.005 | 0.224 |
|  |  |  |  |  |  |  |
| **Unexplained** |  |  |  |  |  |  |
| Age | 7.831 | 4.240 | 1.85 | 0.065 | -0.482 | 16.144 |
| Age squared | -4.959 | 2.215 | -2.24 | 0.025 | -9.300 | -0.617 |
| Married/cohabiting | -0.304 | 0.350 | -0.87 | 0.386 | -0.989 | 0.382 |
| Children in household | -0.653 | 0.310 | -2.10 | 0.035 | -1.261 | -0.045 |
| Limiting long-standing illness | 0.263 | 0.138 | 1.91 | 0.056 | -0.007 | 0.533 |
| Discrimination exposure | -0.159 | 0.146 | -1.09 | 0.277 | -0.446 | 0.128 |
| Log equivalised HH income | 4.009 | 2.156 | 1.86 | 0.063 | -0.219 | 8.236 |
| Employed or student | 0.506 | 0.553 | 0.92 | 0.360 | -0.578 | 1.591 |
| Higher education | -1.167 | 0.289 | -4.04 | <0.001 | -1.734 | -0.601 |
| Home ownership | 1.121 | 0.387 | 2.90 | 0.004 | 0.362 | 1.879 |
| Rural residence | 0.049 | 0.080 | 0.62 | 0.537 | -0.107 | 0.206 |
| Neighbourhood cohesion | -1.505 | 1.638 | -0.92 | 0.358 | -4.717 | 1.707 |
| Constant | -4.796 | 3.342 | -1.43 | 0.151 | -11.348 | 1.757 |

*Married/cohabiting: Legally married or cohabiting with partner*

*Children in household: One or more children aged under 16 in household*

*Limiting long-standing illness: Long-standing health condition that limits daily activities*

*Discrimination exposure: Physically attacked or avoided places due to fear of attack based on ethnicity, religion, or other characteristic*

*Above median income: Equivalised household income above survey-weighted population median*

*Employed or student: Currently in employment or full-time education*

*Higher education: Degree-level qualification or other higher education*

*Home ownership: Owns home outright or with mortgage*

*Rural residence: Rural area (ONS classification)*

*Neighbourhood cohesion: Scale of neighbourhood belonging and trust, 1-5 (carry-forward imputed from rotating module waves)*

*Source: UK Household Longitudinal Study (UKHLS), waves 2-15 (2010-2024). Survey-weighted Oaxaca-Blinder decomposition with pooled coefficients. Linearised standard errors account for complex survey design.*

**Table A24.** Oaxaca-Blinder decomposition of GHQ-12 Likert: ethnic minority women vs rest, with log equivalised household income (replacing the binary above-median indicator)

| **Blinder-Oaxaca decomposition** | | | | | | |
| --- | --- | --- | --- | --- | --- | --- |
| Group 1: Rest of the UK population | | | | Number of observations: 277,896 | | |
| Group 2: Ethnic minority women | | | | Model: Linear (pooled) | | |
| **GHQ-12 Likert** | **Coeff.** | **Linearised SE** | **t** | **P>\|t\|** | **[95% Conf.** | **Interval]** |
| **Overall** |  |  |  |  |  |  |
| Group 1 mean | 11.382 | 0.063 | 181.57 | <0.001 | 11.259 | 11.505 |
| Group 2 mean | 12.089 | 0.197 | 61.29 | <0.001 | 11.702 | 12.476 |
| Difference | -0.707 | 0.203 | -3.48 | <0.001 | -1.105 | -0.309 |
| Explained | -0.432 | 0.081 | -5.33 | <0.001 | -0.591 | -0.273 |
| Unexplained | -0.274 | 0.193 | -1.42 | 0.156 | -0.654 | 0.105 |
|  |  |  |  |  |  |  |
| **Explained** |  |  |  |  |  |  |
| Age | 1.314 | 0.164 | 8.02 | <0.001 | 0.992 | 1.635 |
| Age squared | -1.680 | 0.179 | -9.39 | <0.001 | -2.031 | -1.329 |
| Married/cohabiting | -0.066 | 0.018 | -3.64 | <0.001 | -0.102 | -0.030 |
| Children in household | -0.002 | 0.027 | -0.07 | 0.945 | -0.055 | 0.051 |
| Limiting long-standing illness | 0.147 | 0.042 | 3.51 | <0.001 | 0.065 | 0.229 |
| Discrimination exposure | -0.046 | 0.019 | -2.44 | 0.015 | -0.084 | -0.009 |
| Log equivalised HH income | -0.038 | 0.011 | -3.50 | <0.001 | -0.059 | -0.017 |
| Employed or student | 0.032 | 0.015 | 2.16 | 0.031 | 0.003 | 0.062 |
| Higher education | 0.007 | 0.006 | 1.29 | 0.198 | -0.004 | 0.018 |
| Home ownership | -0.072 | 0.022 | -3.20 | 0.001 | -0.116 | -0.028 |
| Rural residence | 0.035 | 0.024 | 1.48 | 0.138 | -0.011 | 0.081 |
| Neighbourhood cohesion | -0.063 | 0.030 | -2.09 | 0.037 | -0.122 | -0.004 |
|  |  |  |  |  |  |  |
| **Unexplained** |  |  |  |  |  |  |
| Age | -1.847 | 2.440 | -0.76 | 0.449 | -6.631 | 2.937 |
| Age squared | 1.359 | 1.264 | 1.08 | 0.282 | -1.118 | 3.837 |
| Married/cohabiting | 0.206 | 0.201 | 1.02 | 0.306 | -0.189 | 0.601 |
| Children in household | 0.280 | 0.161 | 1.73 | 0.083 | -0.037 | 0.596 |
| Limiting long-standing illness | -0.212 | 0.088 | -2.42 | 0.016 | -0.385 | -0.040 |
| Discrimination exposure | 0.020 | 0.084 | 0.24 | 0.809 | -0.144 | 0.184 |
| Log equivalised HH income | -0.022 | 1.348 | -0.02 | 0.987 | -2.664 | 2.620 |
| Employed or student | -0.228 | 0.290 | -0.78 | 0.433 | -0.797 | 0.342 |
| Higher education | 0.606 | 0.163 | 3.71 | <0.001 | 0.286 | 0.927 |
| Home ownership | -0.647 | 0.227 | -2.85 | 0.004 | -1.092 | -0.202 |
| Rural residence | -0.053 | 0.049 | -1.09 | 0.278 | -0.150 | 0.043 |
| Neighbourhood cohesion | 1.406 | 0.922 | 1.53 | 0.127 | -0.401 | 3.214 |
| Constant | -1.142 | 1.950 | -0.59 | 0.558 | -4.965 | 2.681 |

*Married/cohabiting: Legally married or cohabiting with partner*

*Children in household: One or more children aged under 16 in household*

*Limiting long-standing illness: Long-standing health condition that limits daily activities*

*Discrimination exposure: Physically attacked or avoided places due to fear of attack based on ethnicity, religion, or other characteristic*

*Above median income: Equivalised household income above survey-weighted population median*

*Employed or student: Currently in employment or full-time education*

*Higher education: Degree-level qualification or other higher education*

*Home ownership: Owns home outright or with mortgage*

*Rural residence: Rural area (ONS classification)*

*Neighbourhood cohesion: Scale of neighbourhood belonging and trust, 1-5 (carry-forward imputed from rotating module waves)*

*Source: UK Household Longitudinal Study (UKHLS), waves 2-15 (2010-2024). Survey-weighted Oaxaca-Blinder decomposition with pooled coefficients. Linearised standard errors account for complex survey design.*

**Table A25.** Oaxaca-Blinder decomposition of SF-12 Mental Component: ethnic minority women vs rest, excluding limiting long-standing illness

| **Blinder-Oaxaca decomposition** | | | | | | |
| --- | --- | --- | --- | --- | --- | --- |
| Group 1: Rest of the UK population | | | | Number of observations: 291,711 | | |
| Group 2: Ethnic minority women | | | | Model: Linear (pooled) | | |
| **SF-12 Mental Component** | **Coeff.** | **Linearised SE** | **t** | **P>\|t\|** | **[95% Conf.** | **Interval]** |
| **Overall** |  |  |  |  |  |  |
| Group 1 mean | 47.851 | 0.126 | 380.50 | <0.001 | 47.604 | 48.097 |
| Group 2 mean | 46.019 | 0.372 | 123.64 | <0.001 | 45.289 | 46.749 |
| Difference | 1.832 | 0.389 | 4.70 | <0.001 | 1.068 | 2.595 |
| Explained | 1.656 | 0.162 | 10.19 | <0.001 | 1.337 | 1.974 |
| Unexplained | 0.176 | 0.371 | 0.47 | 0.636 | -0.552 | 0.904 |
|  |  |  |  |  |  |  |
| **Explained** |  |  |  |  |  |  |
| Age | -1.903 | 0.307 | -6.20 | <0.001 | -2.505 | -1.302 |
| Age squared | 3.044 | 0.346 | 8.79 | <0.001 | 2.365 | 3.723 |
| Married/cohabiting | 0.243 | 0.049 | 4.93 | <0.001 | 0.147 | 0.340 |
| Children in household | 0.037 | 0.053 | 0.70 | 0.485 | -0.066 | 0.140 |
| Discrimination exposure | 0.121 | 0.052 | 2.33 | 0.020 | 0.019 | 0.223 |
| Above median income | -0.081 | 0.032 | -2.54 | 0.011 | -0.144 | -0.019 |
| Employed or student | -0.180 | 0.046 | -3.90 | <0.001 | -0.270 | -0.090 |
| Higher education | -0.030 | 0.017 | -1.80 | 0.071 | -0.063 | 0.003 |
| Home ownership | 0.262 | 0.055 | 4.78 | <0.001 | 0.155 | 0.370 |
| Rural residence | -0.032 | 0.046 | -0.70 | 0.483 | -0.122 | 0.058 |
| Neighbourhood cohesion | 0.175 | 0.068 | 2.57 | 0.010 | 0.042 | 0.309 |
|  |  |  |  |  |  |  |
| **Unexplained** |  |  |  |  |  |  |
| Age | 9.800 | 4.670 | 2.10 | 0.036 | 0.645 | 18.955 |
| Age squared | -5.744 | 2.524 | -2.28 | 0.023 | -10.691 | -0.796 |
| Married/cohabiting | -0.513 | 0.380 | -1.35 | 0.178 | -1.259 | 0.233 |
| Children in household | -0.693 | 0.332 | -2.09 | 0.037 | -1.344 | -0.042 |
| Discrimination exposure | -0.056 | 0.169 | -0.33 | 0.740 | -0.387 | 0.275 |
| Above median income | 0.536 | 0.301 | 1.78 | 0.075 | -0.054 | 1.127 |
| Employed or student | 0.046 | 0.674 | 0.07 | 0.946 | -1.277 | 1.368 |
| Higher education | -1.050 | 0.334 | -3.14 | 0.002 | -1.706 | -0.395 |
| Home ownership | 1.137 | 0.437 | 2.60 | 0.009 | 0.279 | 1.994 |
| Rural residence | 0.116 | 0.102 | 1.14 | 0.255 | -0.084 | 0.316 |
| Neighbourhood cohesion | -1.520 | 1.726 | -0.88 | 0.378 | -4.904 | 1.864 |
| Constant | -1.883 | 2.710 | -0.69 | 0.487 | -7.196 | 3.430 |

*Married/cohabiting: Legally married or cohabiting with partner*

*Children in household: One or more children aged under 16 in household*

*Limiting long-standing illness: Long-standing health condition that limits daily activities*

*Discrimination exposure: Physically attacked or avoided places due to fear of attack based on ethnicity, religion, or other characteristic*

*Above median income: Equivalised household income above survey-weighted population median*

*Employed or student: Currently in employment or full-time education*

*Higher education: Degree-level qualification or other higher education*

*Home ownership: Owns home outright or with mortgage*

*Rural residence: Rural area (ONS classification)*

*Neighbourhood cohesion: Scale of neighbourhood belonging and trust, 1-5 (carry-forward imputed from rotating module waves)*

*Source: UK Household Longitudinal Study (UKHLS), waves 2-15 (2010-2024). Survey-weighted Oaxaca-Blinder decomposition with pooled coefficients. Linearised standard errors account for complex survey design.*

**Table A26.** Oaxaca-Blinder decomposition of GHQ-12 Likert: ethnic minority women vs rest, excluding limiting long-standing illness

| **Blinder-Oaxaca decomposition** | | | | | | |
| --- | --- | --- | --- | --- | --- | --- |
| Group 1: Rest of the UK population | | | | Number of observations: 292,586 | | |
| Group 2: Ethnic minority women | | | | Model: Linear (pooled) | | |
| **GHQ-12 Likert** | **Coeff.** | **Linearised SE** | **t** | **P>\|t\|** | **[95% Conf.** | **Interval]** |
| **Overall** |  |  |  |  |  |  |
| Group 1 mean | 11.538 | 0.062 | 185.82 | <0.001 | 11.416 | 11.659 |
| Group 2 mean | 12.347 | 0.216 | 57.29 | <0.001 | 11.925 | 12.770 |
| Difference | -0.810 | 0.221 | -3.67 | <0.001 | -1.242 | -0.377 |
| Explained | -0.537 | 0.079 | -6.76 | <0.001 | -0.693 | -0.381 |
| Unexplained | -0.273 | 0.214 | -1.28 | 0.202 | -0.691 | 0.146 |
|  |  |  |  |  |  |  |
| **Explained** |  |  |  |  |  |  |
| Age | 1.621 | 0.183 | 8.88 | <0.001 | 1.263 | 1.979 |
| Age squared | -1.914 | 0.192 | -9.96 | <0.001 | -2.291 | -1.537 |
| Married/cohabiting | -0.106 | 0.023 | -4.51 | <0.001 | -0.151 | -0.060 |
| Children in household | -0.021 | 0.029 | -0.73 | 0.465 | -0.077 | 0.035 |
| Discrimination exposure | -0.058 | 0.026 | -2.24 | 0.025 | -0.108 | -0.007 |
| Above median income | 0.002 | 0.017 | 0.10 | 0.923 | -0.032 | 0.036 |
| Employed or student | 0.106 | 0.027 | 3.87 | <0.001 | 0.053 | 0.160 |
| Higher education | 0.015 | 0.008 | 1.78 | 0.075 | -0.001 | 0.031 |
| Home ownership | -0.134 | 0.029 | -4.70 | <0.001 | -0.191 | -0.078 |
| Rural residence | 0.045 | 0.025 | 1.80 | 0.071 | -0.004 | 0.093 |
| Neighbourhood cohesion | -0.093 | 0.036 | -2.55 | 0.011 | -0.164 | -0.021 |
|  |  |  |  |  |  |  |
| **Unexplained** |  |  |  |  |  |  |
| Age | -3.594 | 2.637 | -1.36 | 0.173 | -8.764 | 1.575 |
| Age squared | 2.011 | 1.377 | 1.46 | 0.144 | -0.688 | 4.711 |
| Married/cohabiting | 0.403 | 0.226 | 1.79 | 0.074 | -0.039 | 0.845 |
| Children in household | 0.176 | 0.187 | 0.94 | 0.346 | -0.190 | 0.542 |
| Discrimination exposure | -0.062 | 0.095 | -0.66 | 0.512 | -0.248 | 0.124 |
| Above median income | -0.259 | 0.171 | -1.52 | 0.129 | -0.595 | 0.076 |
| Employed or student | 0.046 | 0.339 | 0.14 | 0.891 | -0.617 | 0.710 |
| Higher education | 0.569 | 0.193 | 2.95 | 0.003 | 0.191 | 0.947 |
| Home ownership | -0.627 | 0.252 | -2.48 | 0.013 | -1.122 | -0.132 |
| Rural residence | -0.101 | 0.066 | -1.53 | 0.126 | -0.229 | 0.028 |
| Neighbourhood cohesion | 1.779 | 1.086 | 1.64 | 0.102 | -0.351 | 3.908 |
| Constant | -0.614 | 1.635 | -0.38 | 0.707 | -3.820 | 2.592 |

*Married/cohabiting: Legally married or cohabiting with partner*

*Children in household: One or more children aged under 16 in household*

*Limiting long-standing illness: Long-standing health condition that limits daily activities*

*Discrimination exposure: Physically attacked or avoided places due to fear of attack based on ethnicity, religion, or other characteristic*

*Above median income: Equivalised household income above survey-weighted population median*

*Employed or student: Currently in employment or full-time education*

*Higher education: Degree-level qualification or other higher education*

*Home ownership: Owns home outright or with mortgage*

*Rural residence: Rural area (ONS classification)*

*Neighbourhood cohesion: Scale of neighbourhood belonging and trust, 1-5 (carry-forward imputed from rotating module waves)*

*Source: UK Household Longitudinal Study (UKHLS), waves 2-15 (2010-2024). Survey-weighted Oaxaca-Blinder decomposition with pooled coefficients. Linearised standard errors account for complex survey design.*
